# Metal Organic Framework — Based Mixed Matrix Membranes for Carbon Dioxide Separation: Recent Advances and Future Directions

**DOI:** 10.3389/fchem.2020.00534

**Published:** 2020-07-03

**Authors:** Vengatesan Muthukumaraswamy Rangaraj, Mohammad A. Wahab, K. Suresh Kumar Reddy, George Kakosimos, Omnya Abdalla, Evangelos P. Favvas, Donald Reinalda, Frank Geuzebroek, Ahmed Abdala, Georgios N. Karanikolos

**Affiliations:** ^1^Department of Chemical Engineering, Khalifa University, Abu Dhabi, United Arab Emirates; ^2^Chemical Engineering Program, Texas A&M University at Qatar, Doha, Qatar; ^3^School of Chemistry, Physics and Mechanical Engineering, Faculty of Engineering, Queensland University of Technology, Brisbane, QLD, Australia; ^4^Institute of Nanoscience and Nanotechnology, National Centre of Scientific Research “Demokritos”, Attica, Greece; ^5^Center for Catalysis and Separations (CeCaS), Khalifa University, Abu Dhabi, United Arab Emirates; ^6^ADNOC Gas Processing, Department of Research and Engineering R&D, Abu Dhabi, United Arab Emirates; ^7^Research and Innovation Center on CO_2_ and H_2_ (RICH), Khalifa University, Abu Dhabi, United Arab Emirates; ^8^Center for Membranes and Advanced Water Technology (CMAT), Khalifa University, Abu Dhabi, United Arab Emirates

**Keywords:** membranes, permeability, selectivity, MOF, CO_2_, separation, polymers, mixture

## Abstract

Gas separation and purification using polymeric membranes is a promising technology that constitutes an energy-efficient and eco-friendly process for large scale integration. However, pristine polymeric membranes typically suffer from the trade-off between permeability and selectivity represented by the Robeson's upper bound. Mixed matrix membranes (MMMs) synthesized by the addition of porous nano-fillers into polymer matrices, can enable a simultaneous increase in selectivity and permeability. Among the various porous fillers, metal-organic frameworks (MOFs) are recognized in recent days as a promising filler material for the fabrication of MMMs. In this article, we review representative examples of MMMs prepared by dispersion of MOFs into polymer matrices or by deposition on the surface of polymeric membranes. Addition of MOFs into other continuous phases, such as ionic liquids, are also included. CO_2_ separation from hydrocarbons, H_2_, N_2_, and the like is emphasized. Hybrid fillers based on composites of MOFs with other nanomaterials, e.g., of MOF/GO, MOF/CNTs, and functionalized MOFs, are also presented and discussed. Synergetic effects and the result of interactions between filler/matrix and filler/filler are reviewed, and the impact of filler and matrix types and compositions, filler loading, surface area, porosity, pore sizes, and surface functionalities on tuning permeability are discoursed. Finally, selectivity, thermal, chemical, and mechanical stability of the resulting MMMs are analyzed. The review concludes with a perspective of up-scaling of such systems for CO_2_ separation, including an overview of the most promising MMM systems.

## Introduction

Nowadays, large amounts of CO_2_ are emitted to the atmosphere associated with fossil fuel consumption to fulfill the energy demands for power generation, transportation, industry needs, and other anthropogenic activities, which are generally accepted as the leading cause of climate change and global warming. In the Paris agreement, it was agreed that the temperature rise should be restricted below 2°C, preferably as low as 1.5°C, to limit the detrimental effects of climate change. To reach this goal, society should strive to lower global CO_2_ emissions to near zero by 2050 (Hawes, 2019). One of the ways to reduce these discharge is carbon capture utilization and storage (CCUS), which is still regarded as relatively costly, in particular, due to the cost associated with the capture step. The current CO_2_ capturing at large scale, e.g., from post-combustion mixtures, takes place mostly via regenerative absorption of CO_2_ using solvents such as alkanol amines, which is the primary process of separation of CO_2_ and other contaminants from sources such as natural gas and synthesis gas (Kohl and Nielsen, 1997). Natural gas also often contains other unwanted gas impurities such as hydrogen sulfide (H_2_S), nitrogen (N_2_) etc., which diminish the NG quality, cause corrosion of process units, and result in environmental issues (Abdulrahman and Sebastine, 2013). These components are almost indeed exclusively removed with absorption using alkanol amine solutions for onshore operations.

On the other hand, off-shore operations, particularly in the far East, use membrane technology for the removal of CO_2_ and other impurities from natural gas due to the space and weight limitations of the off-shore sites. In general, for the two applications of natural gas treatment and post-combustion capture, significant research efforts are focusing on improving the existing technologies and developing new routes for CO_2_ removal. Examples of such efforts focus on improved alkanolamines absorption schemes (Vinoba et al., 2013), adsorption (Bhagiyalakshmi et al., 2011; Kueh et al., 2018), chemisorption (Wang et al., 2005; Qaroush et al., 2017), membrane technology (Luis et al., 2012; Adewole et al., 2013; Veziri et al., 2015), cryogenic (Maqsood et al., 2014; Surmi, 2019), and enzymatic (Pierre, 2012; Gundersen et al., 2014) processing.

Both polymeric and inorganic membranes have been used in various industrial applications such as gas separation, microfiltration, reverse osmosis (RO), water purification, heavy metal removal, hemodialysis etc. Indeed, polymeric and inorganic membranes constitute a broad research field for many applications in separation technology. Specifically, for gas separation, the permeance and selectivity coefficients are the main properties that dictate their efficiency and suitability for a specific process. At the same time, porosity, thermal resistance, swelling, and electrical conductivity also play an important role and, depending on the intended application, must be optimized (Labropoulos et al., 2009; Pilatos et al., 2010; Favvas and Papageorgiou, 2013). The advantages of the polymeric membranes include excellent stability, high efficiency, low energy requirement, and ease of operation. Due to these advantages, along with the progress made through R&D activities in academia and industry, several membrane systems have been implemented worldwide over the last decades. Nonetheless, the development of membranes with excellent thermal and mechanical stability combined with good permeability/selectivity properties are essential for industrial integration (Majeed et al., 2012; Stoeger et al., 2012a; Labropoulos et al., 2015).

Membrane technology has intrinsic advantages over the conventional absorption processes due to drawbacks associated with the latter, such as complexity of the process lay-out, corrosivity, and the costly and energy-consuming regeneration (Vinoba et al., 2017). However, a main drawback of polymeric membranes is to withstand higher temperatures, as the latter can be involved in a gas separation process. Recently, thermally stable polymers have received considerable attention in the development of gas separation membranes with durability and process sustainability. For instance, heterocyclic groups incorporated in aromatic polymeric membranes can possess higher thermal and mechanical stability combined with high gas permeance values (Rezakazemi et al., 2018). Membranes have the advantage that at high partial pressure, the flux scales with the concentration. At the same time, amines will get saturated and will require an increasing amount of solvent flow, leading to increased capital and operating cost. Therefore, membrane technology has received promising attention in various industries toward effective CO_2_ separation (Hamid and Jeong, 2018), and it is also potential candidate for dual conversion and capture capability (Perdikaki et al., 2012, 2016). It is worth noting that the Norwegian Government together with Equinor, Shell, and Total started the Northern Lights project for developing an “open source” service for transport and storage of European CO_2_ where the separation activities will play a significant role in the overall objectives (Syversen, 2019).

Generally, inorganic polycrystalline membranes and organic membranes are employed for the CO_2_ separation process, and both systems present advantages but also limitations. Inorganic membranes possess high chemical, thermal, and mechanical stability and long-term durability, and they also exhibit high permeability due to their textural features as compared to polymeric membranes (Pera-Titus, 2014). On the other hand, membrane processability, high cost, brittleness and defects, and scale-up potential, often limit the commercialization of inorganic membranes (Stoeger et al., 2012b; Nasir et al., 2013; Alomair et al., 2015).

Nowadays, the natural gas industry utilizes polymeric membranes for CO_2_ separation due to their inherent flexibility, cost-effectiveness, easy processability, and high durability (Powell and Qiao, 2006; Kim and Lee, 2013). The separation mechanism of polymeric membranes varies based on glassy and rubber content of the polymer matrix. Polymeric membranes with high glassy content separate based on molecular size mainly using diffusion principles (Wang S. et al., 2016), whereas polymeric membranes with high rubber content separate CO_2_ *via* the condensability mechanism (Freeman, 1999). Hence, membrane selectivity and permeability highly depend on the nature of the polymer. Overall, membranes with high glassy content show better CO_2_ selectivity and permeability (Kapantaidakis et al., 1999). However, the polymeric single-phase membranes have the limitation of the permeability-selectivity trade-off issue as depicted in the Robeson plot (Maier, 1998; Robeson, 2008). As a result, significant research has focused on circumventing the “trade-off” problem in the traditional polymeric membranes. Currently, membrane technology development focuses more on the incorporation of inorganic particulates to yield filled hybrid polymer composites membranes, termed as mixed matrix membranes (MMMs) (Chung et al., 2007; Galizia et al., 2017; He et al., 2018). MMMs are composed of homogeneously interpenetrating polymeric and inorganic particles. Specifically, the incorporation of many types of nanomaterials into the membrane's matrices, which often is a complex process, can provide unique properties to the membrane's structure and affect gas transport behavior (Goh et al., 2011). Generally, the most common dispersed inorganic particles in polymer matrices are metal oxides nanoparticles (e.g., MgO and TiO_2_) (Sotto et al., 2011), ZnO (Balta et al., 2012), Al_2_O_3_ (Yu et al., 2011), Au (Vanherck et al., 2011), zero-valent iron (ZVI) (Xu and Bhattacharyya, 2005), Pd (Pacheco Tanaka et al., 2006), magnetic iron-based nanoparticles (Favvas et al., 2019), as well as zeolites (Varoon et al., 2011), carbon molecular sieves (Vu et al., 2003), silicate materials (Zornoza et al., 2011b), non-porous silica (Sanchez et al., 2005), porous silica (Zhang et al., 2008), both multi-walled (MWCNTs) (Favvas et al., 2014), and single-walled (SWCNTs) carbon nanotubes (Das et al., 2014), clays (Zulhairun et al., 2014), and metal-organic frameworks (MOFs) (Rodenas et al., 2014).

MMMs are the alternative to traditional polymeric membranes, as they could rectify the drawback of the “trade-off” effect in commercial membranes. Several factors, such as type and physicochemical properties of fillers, nature of polymer matrix, filler-matrix interaction, processing methods, etc., influence the performance of the MMMs in the CO_2_ separation processes. In particular, the selection of filler material is one of the most crucial parameters for designing efficient MMMs. An appropriate filler selection is challenging because filler materials might exhibit a weak adhesion in the polymer matrix, thus leading to poor CO_2_ separation performance (Dong et al., 2013). Table 1 displays recent developments in the fabrication of MMMs using different porous filler particles exhibiting unique textural and surface properties from porous families such as zeolites (Bastani et al., 2013; Zarshenas et al., 2016), mesoporous silica (Zornoza et al., 2011b; Li et al., 2015), graphene oxide (GO) (Li et al., 2015; He et al., 2019), CNTs (Bakhtin et al., 2015; Dai et al., 2019), and MOFs (Safak Boroglu and Yumru, 2017; Etxeberria-Benavides et al., 2018). Among these fillers, MOFs have received considerable attention, and numerous MOFs and their hybrids have been utilized as the dispersive fillers to fabricate efficient MMMs for CO_2_ separation. This review provides insights into the role and advantages of MOFs as well as hybrid and functionalized MOFs in MMMs for CO_2_ separation.

**Table 1:** CO_2_ separation performance of MMMs using different porous fillers.

**Polymer**	**Filler**	**P (bar)**	**T (^**°**^C)**	**CO_**2**_ permeability (Barrer)**	**Selectivity CO_**2**_/CH_**4**_ CO_**2**_/CH_**4**_**	**Selectivity CO_**2**_/N_**2**_**	**References**
Polyurethane	Zeolite-4A (12 wt. %)	10	25	81.63	7.38	23.6	Afarani et al., 2018
Matrimid® 5218	Li/Na-ZSM-25 (5 wt. %)	5	35	12	169	–	Zhao J. et al., 2019
Matrimid®5218	NaY zeolite (15 wt. %)	2	35	17.52	43.3	–	Ebadi Amooghin et al., 2015
Matrimid®5218	Sm-NaY (15 wt. %)	2	35	9.7	57.1	–	Ebadi Amooghin et al., 2015
Pebax-1657	Zeolite-4A (30 wt. %)	3.75	25	155.8	7.9	–	Surya Murali et al., 2014
Polysulfone	MCM-41-NH_2_ (20 wt. %)	1	25	7.89	–	41.52	Miricioiu et al., 2019
Polyurethane	MCM-48 (20 wt. %	10	25	70.2	4.5	21.1	Ghalei et al., 2018
Polyimide	Acid-treated MWCNTs (3 wt. %)	1	15	9.06	24.49	37.74	Sun et al., 2017
Polyimide	Chitosan functional MWCNT (1 wt. %)	15	25	37.31	16.5	–	Aroon et al., 2010
PIM-1	PEG functionalized MWCNT (3 wt. %)	2	30	4,816	16.3	22.2	Khan et al., 2013
Polyvinylalcohol matrix containing amines	MWCNT (2 wt. %)	15.2	107	856	–	–	Zhao et al., 2014)
Pebax	Porous r-GO oxide (5 wt. %)	2	30	119	–	104	Dong G. et al., 2016
Polyimide	Amine-functional GO (3 wt. %)	1	15	12.34		38.56	Ge et al., 2018
Pebax MH 1657	PEG–PEI–GO (10 wt. %)	2	30	1,330	45	120	Li et al., 2015
Pebax-1074	ZIF-7 (10 wt. %)	3	30	123	24	–	Azizi and Hojjati, 2018
Matrimid®-PEG 200	ZIF-8 (30 wt. %)	8	25	31.4	15.42	–	Castro-Muñoz and Fíla, 2019
Polysulfone	NH_2_-MIL-125(Ti) (20 wt. %)	3	29.8	30	29.5		Guo et al., 2015
Polyimide	UiO-66 (17 wt. %)	2	35	108	41.9	–	Zamidi Ahmad et al., 2018
Polysulfone	Bio-MOF-1 (30 wt. %)	10	25	16.57	42.6	45.6	Ishaq et al., 2019
Cellulose acetate	NH_2_-MIL-53(Al)	3	25	52.6	28.7	23.4	Mubashir et al., 2018b
6FDA-DAM polymer	ZIF-94 (40 wt. %)	1	25	2,310	–	22	Etxeberria-Benavides et al., 2018
6FDA-DAM polymer	ZIF-11(20 wt. %)	4	30	257.5	31.02	–	Safak Boroglu and Yumru, 2017

## Significance of MOFs in MMMs

Currently, MMMs are effectively contributing to membrane technology for CO_2_ removal due to their flexibility and multifunctionality. These composite membranes are developed by incorporation of different types of inorganic fillers (porous and non-porous) into the polymer matrices mainly through solution blending, *in situ* polymerization, and sol-gel processing (Cong et al., 2007). The dispersed fillers can change the diffusion pathway, free volume, and mechanical and thermal properties of the polymer matrix. MMMs containing non-porous fillers typically provide only selective adsorption of particular molecular species, whereas, porous fillers can directly change the MMMs permeability and selectivity based on their porous network within the membrane (Goh et al., 2011; Zhang et al., 2013; Salehi Maleh and Raisi, 2019). A small fraction of inorganic nanofillers can effectively interact with the polymer matrix, e.g., through hydrogen bonding, Van der Waals or covalent bond interactions, which also enhance the chemical, mechanical and thermal stability of the polymeric membranes (Siddique et al., 2014). Combining organic polymers with inorganic fillers can yield MMMs exhibiting significant enhancement of CO_2_ separation, and it can also effectively help overcome the “trade-off” issues existing in pure polymeric or inorganic membranes (Althues et al., 2007; Mubashir et al., 2018a). Nonetheless, the filler dispersion in the polymer matrix plays a predominant role to acquire defect-free MMMs. Besides, the chemical structure, surface functional groups, aspect ratio, and textural properties are essential factors for the proper dispersion of the filler materials. It is worth noting that surface-modified inorganic fillers lead to a robust interfacial adhesion with the polymer matrix, resulting in effective dispersion and formation of defect-free MMMs (Guo et al., 2015). Though both zeolites and MOFs are actively used as dispersive fillers, the advantages of MOFs include higher porosity, availability in various sizes and shapes, and presence of the organic part which enhances compatibility with the polymer matrix.

MOFs have gained significant interest in various fields, including catalysis, separation, purification, biomedicine, sensors, microelectronics, etc. (Keskin and Kizilel, 2011; Falcaro et al., 2014; Mubashir et al., 2018b; Wang B. et al., 2018). MOFs consist of metal ions or metal-oxide clusters combined with organic ligands, and are highly porous, often hierarchical, chemically flexible, while they can possess multiple chemical architectures (Li Z. et al., 2019; Safaei et al., 2019). MOFs have been recognized as attractive filler materials for the development of MMMs owing to their inherent three-dimensional (3D) coordination network with effective porosity and good interaction with the continuous polymer phase (Cheng et al., 2019). Manipulating the organic ligands and the surface of the MOFs with suitable chemical functionalities gives rise to the formation of microvoids between the organic and inorganic phases and minimizes the selectivity loss (Ordoñez et al., 2010; Dai et al., 2012; Sorribas et al., 2014). Compared to MOFs, other dispersive fillers often have limited chemical flexibility and mutability, which may restrain their dispersion within the polymer matrix. In addition, the MOF frameworks are chemically flexible. Thus they can contribute to selective adsorption of certain molecular species from the gaseous mixture and enhance the separation performance of the membranes (Dechnik et al., 2017). Based on the type and structure of the polymer matrix, a wide range of MOFs are available to be used as fillers with a variety of organic functionalities and chemical flexibility of the organic part of the framework. Besides, suitable post-synthetic modification of MOFs can significantly enhance the polymer-MOF interfacial interaction and increase the separation efficiency (Katayama et al., 2019). Recently, MMMs have also been developed *in situ* by growing MOFs within the polymer matrix followed by curing of the latter. The as-prepared MMMs often show high separation efficiency as compared to the post-synthesis MMMs (Marti et al., 2018). In addition, MOFs are easily hybridized with other porous filler materials such as CNTs, GO, and mesoporous silica, owing to their intrinsic chemical functionality, which provides a synergetic effect with the other fillers within the polymer matrix (Pilatos et al., 2014; Lin et al., 2015; Naik et al., 2016; Anastasiou et al., 2018; Pokhrel et al., 2018b). Most MOF fillers exhibit higher porosity than zeolites and other porous materials. They can be more compatible with polymer matrices, while tuning of their textural properties can also offer tunability for permeability and selectivity in the resulting MMMs (Janiak and Vieth, 2010).

## MOF Based MMMs for CO_2_ Separation

### Pristine MOF—Based MMMs

Numerous MOFs have been developed and studied for various applications (Furukawa et al., 2013), however, only a rather small portion of those as of now, such as HKUST-1, zeolitic imidazole frameworks (ZIFs), MILs, MOF-74, and UiO-66, have been investigated as filler for MMMs. These MOFs are ideal materials for enhancing the membrane performance due to their tunable porosity, gas selectivity and permeability, solubility and diffusivity, and strong interactions with the polymer matrix as to yield defect-free MOF-based MMMs (Erucar et al., 2013; Chen K. et al., 2018; Fan L. et al., 2018; Nabais et al., 2018; Zhao Y. et al., 2019). Nabais et al. fabricated MMMs using Fe(BTC) and Matrimid®5218 for CO_2_ separation (Nabais et al., 2018). The filler loadings were varied from 0 to 30 wt. % and resulted in enhancing thermal stability and hydrophobicity of the MMMs. Generally, the CO_2_ permeability increases with the addition of MOF, yet the extent of the permeability increase depends on the nature of MOFs and the degree of functionalization. Contrary to the behavior of other membranes, the selectivity of the MMMs significantly increased with increasing the temperature from 29 to 78°C in that work (Table 2). This is attributed to the higher mobility of the polymeric chains at the higher temperatures and increased free volume that enhance gas diffusivity (Ilyas et al., 2017). Moreover, the MMMs containing 10 and 30 wt. % Fe(BTC) demonstrated better performance in CO_2_/N_2_ separation as compared to other commercial membranes.

**Table 2:** Effect of temperature on the CO_2_ permeability and CO_2_/N_2_ ideal selectivity of the Matrimid®5218 based MMMs consisting of 10, 20, and 30 wt. % of Fe (BTC) (Nabais et al., 2018).

**T (^**°**^C)**	**30**		**50**		**80**	
**MOF loading (wt. %)**	**CO_**2**_ permeability (Barrer)**	**Selectivity CO_**2**_/N_**2**_**	**CO_**2**_ permeability (Barrer)**	**Selectivity CO_**2**_/N_**2**_**	**CO_**2**_ permeability (Barrer)**	**Selectivity CO_**2**_/N_**2**_**
0	9.6 ± 0.1	24.0 ± 0.2	12.4 ± 0.1	9.0 ± 0.2	14.6 ± 0.1	4.4 ± 0.2
10	29.8 ±0.7	7.5 ± 0.8	48.3 ± 0.1	10.1 ± 0.2	84.9 ± 0.1	43.5 ± 0.3
20	77.2 ± 0.1	6.1 ± 0.2	91.9 ± 0.1	5.7 ± 0.1	91.2 ± 0.2	15.4 ± 0.4
30	94.2 ± 0.5	6.0 ± 0.6	109.6 ± 0.1	4.7 ± 0.2	217.9 ± 0.2	23.1 ±0.3
α^ads^ (1 bar)	9.2		7.6		5.5	

**Figure 1:**
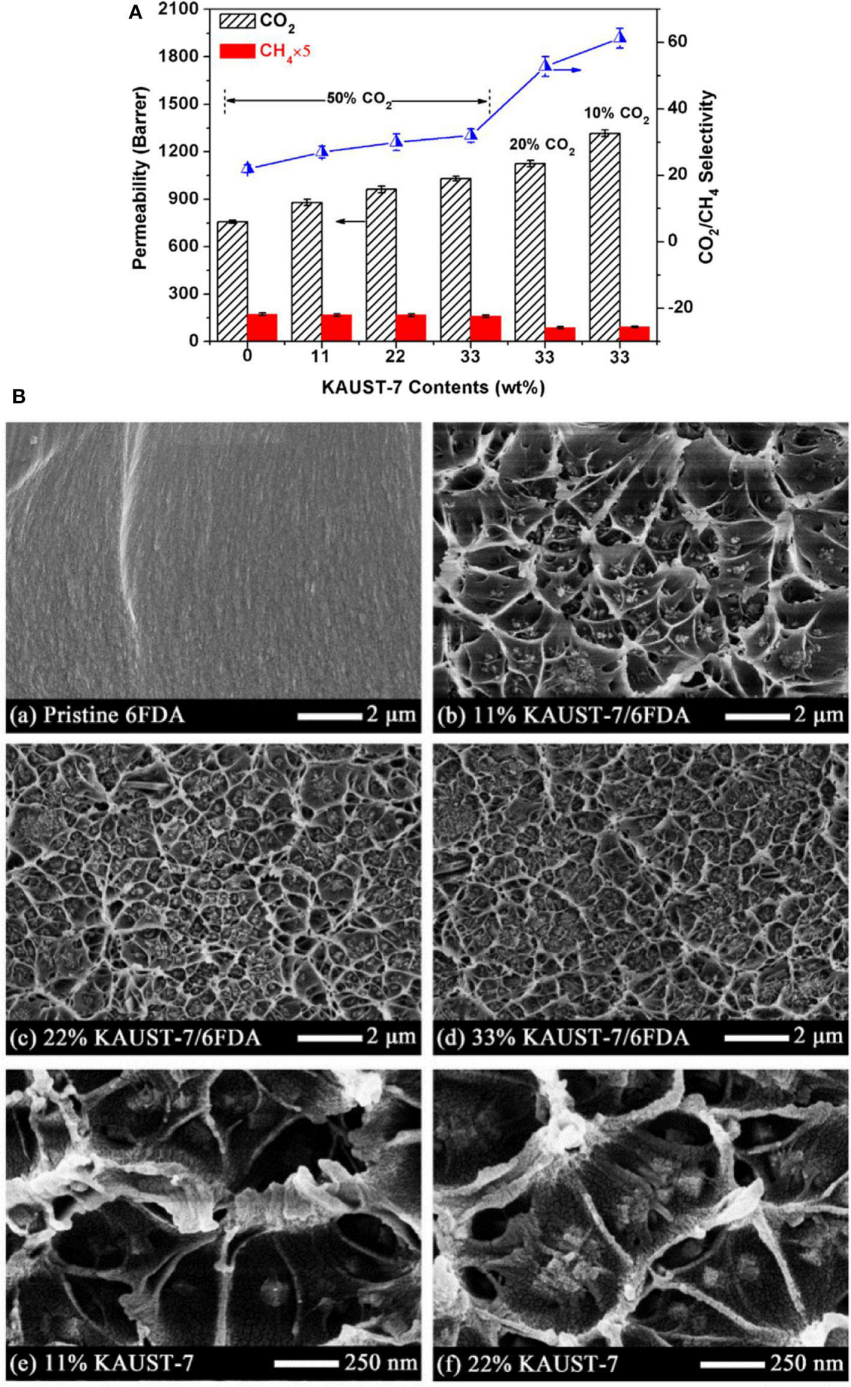
**(A)** CO_2_ separation efficiency of 6FDA-Durene based MMMs with various KAUST-7 loadings at 35°C and 2 bar (% is CO_2_ concentrations in the feed gas). **(B)** SEM cross-section images of pristine 6FDA membrane and 6FDA based MMMs with wt. % contents of KAUST-7 nanocrystals (~80 nm). Reproduced from Chen K. et al. (2018) with permission from Elsevier.

Zhao et al. fabricated MMMs using NH_2_-CAU-1 with polyimide matrix-supported on porous ceramic membrane *via* solvent casting, and the prepared MMMs demonstrated excellent separation performance in terms of permeation rate and selectivity. 20 wt. % NH_2_-CAU-1 was the optimum loading as revealed by the significant enhancement in the H_2_/CO_2_ selectivity compared to the selectivity of 6.5 for the pristine membrane (Zhao Y. et al., 2019). In another study, Fan et al. incorporated two types of MOFs, i.e., Ni_2_(L-asp)_2_bipy and Ni_2_(L-asp)_2_pz with different pore sizes into poly(ether-block-amide) (Pebax-1657) and the MMM consisting of 20 wt. % of Ni_2_(L-asp)_2_bipy showed maximum CO_2_ permeability of 120 Barrer compared to the pristine membrane (55 Barrer) (Fan L. et al., 2018). Chen et al. also reported enhancement in the performance of MMMs using KAUST-7 as the nano-filler with narrow size distribution prepared by co-solvent synthesis (Chen K. et al., 2018). Both the CO_2_/CH_4_ selectivity and CO_2_ permeability increased with increasing filler content in the MMM (Figure 1A). The improved performance of the MMMs is partially attributed to the strong interfacial interaction between the filler and the polymer, as can be seen in the SEM images in Figure 1B, where a circular network morphology is recognized (Chen K. et al., 2018).

**Figure 2:**
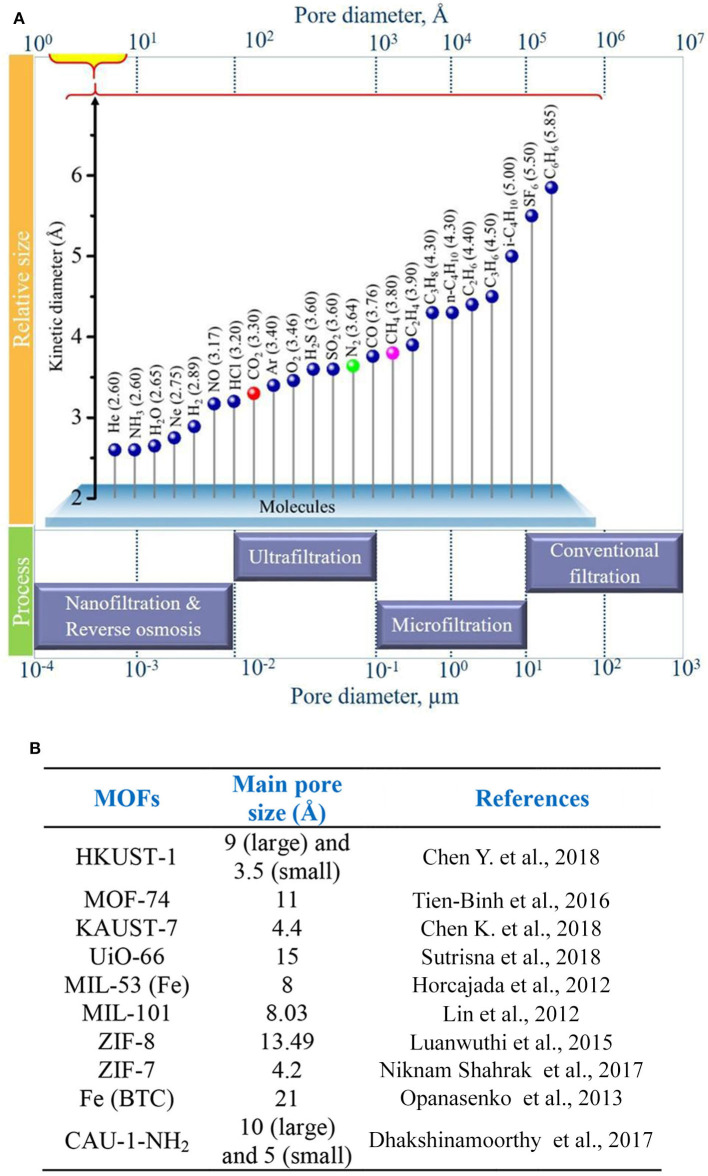
**(A)** Relationships between pore diameter of fillers and kinetic diameter of gas molecules for different separation processes. Reproduced from Vinoba et al. (2017) with permission from Elsevier. **(B)** Types of MOFs with respective critical pore sizes.

Cheng et al. prepared ultrathin MMMs using copper 1,4-benzenedicarboxylate nanosheets (CuBDC) and polymers of intrinsic microporosity (PIMs) via spin coating, and they investigated the impact of the membrane thickness and CuBDC loadings on the performance of the MMMs. An optimum filler loading of 10 wt. % of CuBDC comprising MMM with a thickness of 660 nm showed CO_2_/CH_4_ selectivity of 15.6 and CO_2_ permeance of 407 GPU. Furthermore, the stability of the membranes was also studied by carrying out analysis for 100 h, confirming the high stability of the prepared MMM (Cheng et al., 2017). Chi et al. expanded the study of MMMs based on multidimensional MOFs to synergize the advantages of different filler dimensions. A mechanism to form a multidimensional HKUST-1 filler was proposed, based on using a modulator to adjust the MOF nucleation and growth. As a result, a 2.5-fold increase in CO_2_ permeability was obtained with the MMM containing 30 wt. % of the multidimensional MOF compared to that of the pristine polymer (Chi et al., 2019). Table 3 compares the CO_2_ separation performance of the above mentioned pristine MOF—based MMMs. MMMs based on hierarchical fillers such as zeolitic type MOF have also received significant attention due to their separation efficiency (Sridhar et al., 2007; MacDowell et al., 2010; Rezakazemi et al., 2014; Alqaheem et al., 2017; Vinoba et al., 2017). In general, several targeted functional groups such as dimers, trimers, tetramers or polyhedral chains can be easily incorporated into MOFs as functional linkers that can be further modified with various organic groups to control the final properties. In general, molecular diffusion mechanism depends on the size of the gas molecule. Thus, the pore diameter is a crucial factor for CO_2_ separation (Mao and Sinnott, 2001; Sridhar et al., 2007; Du et al., 2012; Alqaheem et al., 2017). Figure 2A shows the relationship between the pore diameter of fillers and various gas molecules for their efficient separation. In addition, different types of MOF fillers together with their respective characteristic pore sizes are shown in Figure 2B. Critical factors such as porosity, pore size, tortuosity, particle size, affinity of MOF fillers with the gas molecules and the matrix, MOF type, geometry, functional groups, and percentage loading of filler influence the transport behavior through the porous membranes (Aykac Ozen and Ozturk, 2019). For instance, small pore sizes of the MOF fillers can selectively allow small molecules to permeate, such as H_2_, He, and CO_2_ with kinetic diameters of 2.6, 2.89, and 3.3 Å, respectively, while larger pore size of MOFs can allow transport of larges gas molecule like CH_4_ (kinetic diameter = 3.8 Å), thus resulting in tailored selectivity (Cheng et al., 2017).

**Table 3:** CO_2_ separation performance of MMMs using selected pure MOF fillers.

**Polymer**	**Fillers (wt. %)**	**T (^**°**^C)**	**P (bar)**	**CO_**2**_ permeability (Barrer^**a**^/GPU^**b**^)**	**Selectivity**		**References**
					**Value**	**Relative to**	
Pebax-1657	Ni_2_ (L-asp)_2_bipy; Ni_2_(L-asp)_2_pz (20 wt. %)	35	1	120.2^a^	32.88	CO_2_/H_2_	Fan L. et al., 2018
6FDA-Durene	KAUST-7 (33 wt. %)	35	2	1,030^a^	19.7	CO_2_/CH_4_	Chen K. et al., 2018
PIMs	CuBDC (10 wt. %)	25	1	407^b^	15.6	CO_2_/CH_4_	Cheng et al., 2017
Pebax-1657	UiO-66-NH_2_ (50 wt. %)	25	2	338^b^	57	CO_2_/N_2_	Sutrisna et al., 2018
Pebax	ZIF-8 (90 nm) (5 wt. %)	20	1	99.7^a^	59.6	CO_2_/N_2_	Zheng et al., 2019
PIMs	MOF-74 (20 wt. %)	25	2	21,269^a^	28.7	CO_2_/N_2_	Tien-Binh et al., 2016
Matrimid®5218	Fe (BTC) (30 wt. %)	80	1	217.9^a^	23.1	CO_2_/N_2_	Nabais et al., 2018
PI	NH_2_-CAU-1(20 wt. %)	23	2	0.94^a^	32.8	H_2_/CO_2_	Zhao Y. et al., 2019
PI	Multidimensional HKUST-1 (30 wt. %)	35	1	2,500^a^	16	CO_2_/N_2_	Chi et al., 2019

#### UiO-66 Based MMMs

**Figure 3:**
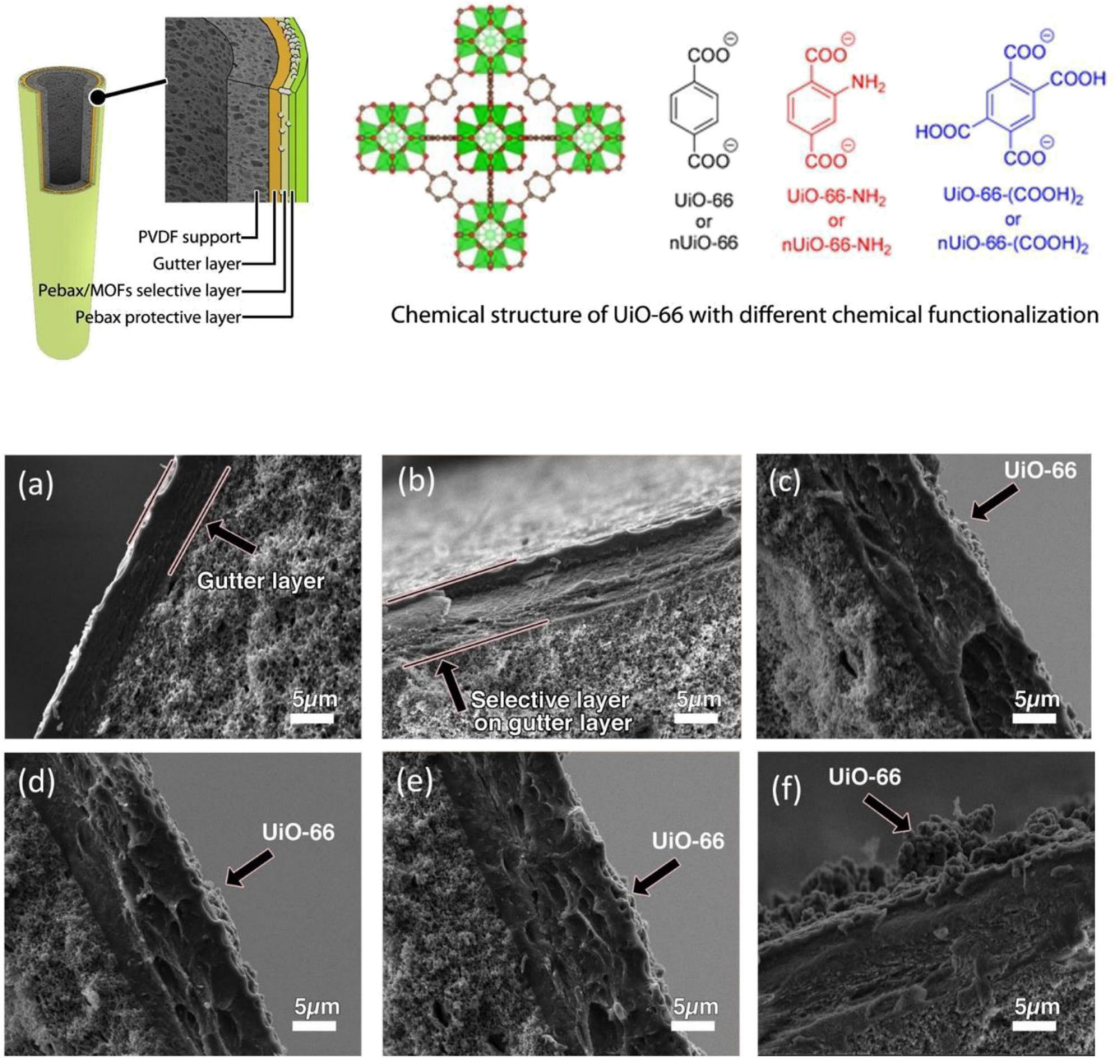
Schematic representation of a UiO-66 MMM and of different chemical functionalization schemes for UiO-66, and effect of different coating layers on the membranes: **(a)** PTMSP, **(b)** PTMSP and pure Pebax, **(c)** PTMSP and 50 wt. % UiO-66 in Pebax, **(d)** PTMSP and 50 wt. % UiO-66-NH_2_ in Pebax, **(e)** PTMSP and 50 wt. % UiO-66-(COOH)_2_ in Pebax and **(f)** PTMSP and 80 wt. % UiO-66 in Pebax. Membranes **(b–f)** have an extra top protective layer. Reproduced from Sutrisna et al. (2018) with permission from RSC.

UiO-66 constitutes a zirconium-based framework bearing carboxylate linkers and exhibits high thermal stability (up to 550°C) and excellent chemical stability toward the water and organic solvents (Moghaddam et al., 2018). UiO-66 with amine (UiO-66, UiO-66-NH_2_), and carboxylic (UiO-66-(COOH)_2_) functional groups (Figure 3) were prepared by Sutrisna et al. and utilized as fillers for the fabrication of MMMs (Sutrisna et al., 2018). The amine-functionalized UiO-66 significantly improved the perm-selectivity of the MMMs. Figure 3 shows SEM images of the cross-sectional area of different MMMs prepared and the effect of the various coating layers from a microscopic point of view. Poly [1-(trimethylsilyl) prop-1-yne] (PTMSP) possessed a high perm-selectivity value. It was used as a smooth gutter layer, which prevented the intrusion of PEBAX into the supportive membrane pores, forming a thin and continuous selective layer. Moreover, the incorporation of UiO-66 resulted in higher stability of the resulting MMMs under high pressure.

Other researchers study the use of hydroxylated (-OH), amino (-NH_2_), nitro (-NO_2_), and methoxy (-OMe) functionalized UiO-66 structures to investigate the effect of a polar and basic functional group on CO_2_ separation. Functionalized UiO-66 to exhibit polar surfaces have been shown to improve CO_2_ adsorption and are attractive for increasing CO_2_/CH_4_ and CO_2_/N_2_ selectivity (Cmarik et al., 2012; Biswas and Van Der Voort, 2013; Wu et al., 2013). Smith et al. modified UiO-66 with titanium (Ti) *via* post-ligand exchange functionalization, and the Ti-exchanged UiO-66 material was mixed with PIM-1 matrix, yielding MMMs with enhanced CO_2_ permeability of 8,200 Barrer and a minor loss of selectivity (Smith et al., 2015). The improved permeability was strongly attributed to the interaction between the filler and polymer matrix, which led to the formation of interfacial free volume and a strong affinity with CO_2_. Meanwhile, a series of MMMs have been fabricated using UiO-66 with a variety of functional groups, with the latter strongly influencing the CO_2_ permeability and selectivity in the resulting MMM (Anjum et al., 2015; Smith et al., 2015; Venna et al., 2015; Khdhayyer et al., 2017).

#### ZIF—Based MMMs

**Figure 4:**
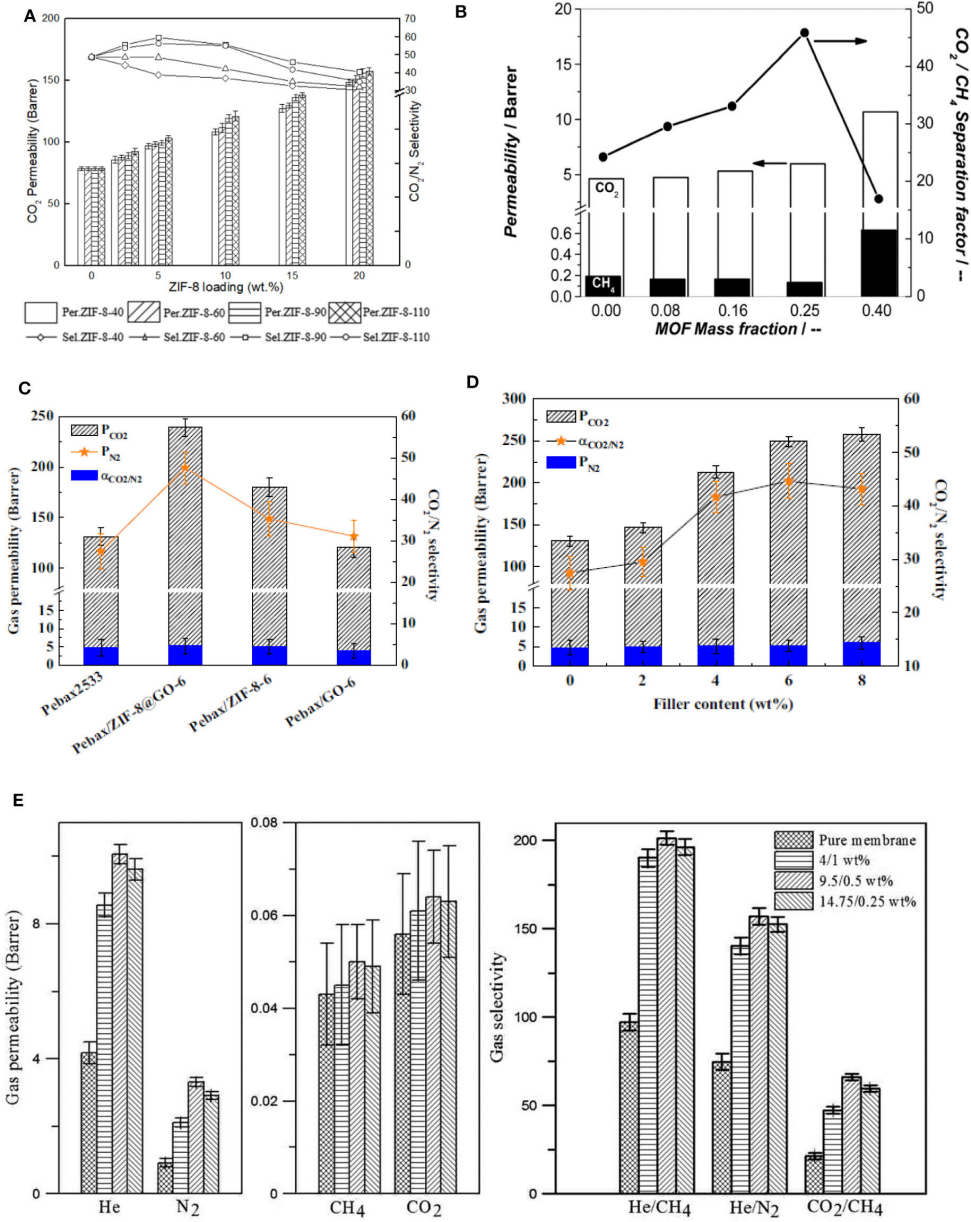
Characteristic examples of MOF filler effects: **(A)** Effect of ZIF-8 loading and size on CO_2_ permeability and CO_2_/N_2_ selectivity of Pebax-ZIF-8 MMMs. Reproduced from Zheng et al. (2019) with permission from Elsevier. **(B)** Separation performance of various NH_2_-MIL-53(Al) MMMs with different MOF loadings at 308 K for a mixture CO_2_:CH_4_ = 1:1. *P*_perm_ = 1 bar, *P*ret = 4 bar. CH_4_ (black bars) and CO_2_ (white bars) permeability (left y-axis) and CO_2_/CH_4_ separation factor (right y-axis). 1 Barrer = 3.348 × 10^−19^ kmol m/(m^2^ s Pa). Reproduced from Zornoza et al. (2011a) with permission from RSC. **(C)** Effect of different Pebax fillers, namely, MMMs with ZIF-8@GO, ZIF-8 and GO, and **(D)** filler content in Pebax/ZIF-8@GO MMMs. Reproduced from Dong et al. (2016a) with permission from Elsevier. **(E)** Gas permeability and selectivity of MMMs with different CuBTC/GO loadings. Reproduced from Feijani et al. (2018) with permission from RSC.

Zeolitic imidazolate frameworks (ZIFs) is a subfamily of MOFs with a structure resembling that of zeolites. ZIFs exhibit tunable properties and possess high thermal and chemical stability due to the synergistic effect of the metal and imidazole groups. Phan et al. have reviewed ZIF based materials for CO_2_ capture and separation (Phan et al., 2010). ZIFs are made of metal nodes connected to organic imidazole linkers that replace the bridging oxygen of zeolite (Kontos et al., 2014). Meanwhile, various ZIF structures with different organic linkers have been employed as fillers for preparing MMMs. ZIFs can exhibit large cavities and narrow pore size, making them suitable as fillers for gas selective MMMs, particularly for CO_2_ separation, because the kinetic diameter of CO_2_ (3.3 Å) is comparable to the pore openings in several ZIFs. Also, ZIFs having the flexibility to adapt a second metal in their structure offer a synergetic effect that can result in higher performance gas separation in MMMs (Mirqasemi et al., 2020). Among the different polymers, Pebax is one of the most promising polymeric matrices due to its good characteristics in terms of flexibility, durability and thermo-mechanical properties. Also, Pebax possesses good selectivity toward polar–non-polar gases, such as CO_2_/N_2_. Various ZIFs have been incorporated into Pebax and other polymers to prepare MMMs (Phan et al., 2010; Kontos et al., 2014; Vinoba et al., 2017; Zheng et al., 2019). For example, MMMs were fabricated using ZIF-8 nanoparticles and poly (ether-block-amide) Pebax matrix. A microemulsion method was used for the preparation of the ZIF-8 nanoparticles with different sizes of 40, 60, 90, and 110 nm (ZIF-8-40, ZIF-8-60, ZIF-8-90, and ZIF-8-110) (Zheng et al., 2019). In this work, it was observed that ZIF-8 with smaller sizes possess a strong affinity with the polymer matrix resulting in strong interfacial interactions and defect-free MMMs. Figure 4A shows the effect of ZIF-8 particle size on the gas permeability and selectivity of PEBAX-based MMMs. MMM with 5 wt. % ZIF-8 (90 nm size) shows an increase in both CO_2_ permeability and CO_2_/N_2_ selectivity as compared to the pure Pebax membrane due to increase in the free volume of the polymer facilitated by the larger ZIF-8 particles. However, the CO_2_/N_2_ selectivity slightly decreased due to the microphase separation in the MMMs. The size of the ZIF-8 particles strongly influences the separation efficiency of MMMs (Zheng et al., 2019).

Castra-Munoz et al. fabricated MMMs using Matrimid®-PEG 200 matrices and ZIF-8, and incorporation of 30 wt. % of ZIF-8 *via* the traditional method increased the CO_2_ permeability to 130 Barrer. On the other hand, the MMM containing 30 wt. % ZIF-8 *via* the non-dried MOF method resulted in higher CO_2_/CH_4_ selectivity of 15 as compared to the MMM prepared by the traditional method (Castro-Muñoz and Fíla, 2019). Benavides et al. prepared high-performance MMMs using ZIF-94 and 6FDA-DAM polymer, and the MMM containing 40 wt. % of ZIF-94 showed the highest CO_2_ permeability of 2,310 Barrer with the CO_2_/N_2_ selectivity of 22 at 1 bar (Etxeberria-Benavides et al., 2018). Li et al. developed MMMs employing porous polyacrylonitrile (PAN) support and using small size ZIF-7 (30–35 nm), Pebax®1657 as the matrix, and PTMSP as the gutting layer to obtain MMMs with a smooth surface. At a lower filler content, both CO_2_ permeability and selectivity (CO_2_/CH_4_ and CO_2_/N_2_) of the MMM was higher than those of the pristine membrane. However, higher loading (34 wt. %) of ZIF-7 increased CO_2_/CH_4_ selectivity to 44, but reduced the permeability compared to pure Pebax®1657, because the higher ZIF-7 filler loading induced polymer rigidification (Li et al., 2013). Based on these studies, the overall MMM performance is affected by filler type, filler size, filler loading, and incorporation method (Phan et al., 2010; Sorribas et al., 2014; Thompson et al., 2014; Nordin et al., 2015; Dong et al., 2016a; Tanh Jeazet et al., 2016). Interestingly, ZIF-ionic liquid (IL) composite membranes have also been prepared to take advantage of the inherent affinity of several ILs with CO_2_ (Tzialla et al., 2013).

#### MIL—Based MMMs

MIL (Materials Institute Lavoisier) is another series of MOFs consisting of organic functional linkers connected with metal ions such as Al, Cr, Ti by corner-sharing. These MOFs possess large pore volume and surface area, and good adsorption capacity, while their framework is comprised of unidirectional, diamond-shaped pore channels. Recently, MILs have been used as fillers for the fabrication of MMMs due to their uniform dispersion and excellent compatibility with various polymers that make them suitable for gas separation. MILs can exist at multiple structures, namely MIL-53(Al), MIL-68(Al), MIL-101(Cr), and MIL-125(Al) (Rezakazemi et al., 2014; Vinoba et al., 2017). Many MILs have been prepared and used as fillers for the fabrication of MMMs (Ordoñez et al., 2010; Opanasenko et al., 2013; Luanwuthi et al., 2015; Niknam Shahrak et al., 2017; Chen Y. et al., 2018). Dorosti et al. fabricated MMMs using Matrid® polymer with different loadings (0–20 wt. %) of MIL-53 (Dorosti et al., 2014). The carboxylate linkage in the MIL-53 possesses a strong affinity with the Matrid® polymer and improves the interfacial adhesion, which resulted in a high separation performance of the MMMs. Individually, MMM containing 15 wt. % of MIL-53 exhibited a CO_2_/CH_4_ selectivity of 51.8 compared to 28.2 for the pure Matrid® membrane. Zornoza et al. fabricated MMMs using polysulfone and amine-functionalized (NH_2_-MIL-53(Al)) MIL. The addition of 25 wt. % of MIL into the polysulfone matrix enhanced CO_2_ transport and decreased the CH_4_ permeation, resulting in a significant increase of CO_2_/CH_4_ selectivity (Figure 4B). Notably, the optimum loading of the majority of inorganic fillers cannot exceed ~10 wt. % due to the mediocre filler–polymer adhesion/interaction (Zornoza et al., 2011a). For example, higher loading of amino-functionalized MOF suppressed the performance of polysulfone based MMMs, due to the formation of week hydrogen bonding between the amine and sulfone groups (Zornoza et al., 2011a).

#### MOF-74—Based MMMs

MOF-74 or M^+^/dobdc, where “M” is the identity of the metal node (Mg, Ni, Co, Zn etc.,), consists of metal ions linked together by 2,5 -dioxido-1,4-benzenedicarboxylate (dobdc) linkers, resulting in a framework of one-dimensional hexagonal morphology with uniform pores in a range of 3–10 Å (Suh et al., 2017). MOF-74 structures are used as adsorbents with high CO_2_ adsorption capacity as well as fillers for the fabrication of MMMs, resulting in enhanced CO_2_ separation performance.

Bachman et al. prepared MMMs using various polymer matrices such as cellulose acetate, commercial Matrimid@, 6FDA-DAT, 6FDA-DAM-DAT, 6FDA-DAM, 6FDA-durene with different loadings of Ni_2_(dobdc) (15–23 wt. %) (Bachman and Long, 2016). Ni(dobdc) possess a strong interaction with the polymer and substantially reduces the plasticization of the MMMs. In particular, the 6FDA-DA/15 wt. % Ni_2_(dobdc) exhibited the highest selectivity of 51.9 by retaining a permeability of 63.9 Barrer. Bae et al. developed MMMs using Mg_2_(dobdc) nanocrystals with the glassy polyimide and rubbery polymers. Glassy polyimide-baseded MMMs showed a significant increment in both the CO_2_ permeability and CO_2_/N_2_ selectivity. However, MMMs made with rubbery polymers exhibited a low permeability due to plugging of the outer pore windows of the Mg_2_(dobdc) pores by the rubbery polymer chains, which have high mobility at room temperature (Bae and Long, 2013). The MOF-74 loading plays a significant role in permselectivity of the MMMs, especially for the CO_2_/CH_4_ system (Bachman and Long, 2016; Li W. et al., 2019). Tien-Binh et al. (2016) prepared MMMs using PIM-1 and different loading of Mg(MOF-74) and the 20 wt. % of MOF-74 filler resulted in a defect-free MMM with CO_2_ permeability of 21.3 Barrer and CO_2_/CH_4_ selectivity of 19.1. Table 4 shows the CO_2_ separation efficiency of MOF-74 based MMMs.

**Table 4:** MOF-74—based MMMs for CO_2_ separation.

**Polymer**	**Filler**	**P (bar)**	**T (^**°**^C)**	**CO_**2**_ permeability (Barrer)**	**Selectivity CO_**2**_/CH_**4**_ CO_**2**_/CH_**4**_**	**Selectivity CO_**2**_/N_**2**_**	**References**
Cellulose acetate	Ni_2_(dobdc) 23 wt. %	1	35	3.78	30.3	–	Bachman and Long, 2016
Matrimid^@^	Ni_2_(dobdc) 23 wt. %	1	35	9.31	29.5	–	Bachman and Long, 2016
6FDA-DAT	Ni_2_(dobdc) 15 wt. %	1	35	63.9	51.9	–	Bachman and Long, 2016
6FDA-DAM-DAT	Ni_2_(dobdc) 18 wt. %	1	35	220	30.5	–	Bachman and Long, 2016
6FDA-DAM	Ni_2_(dobdc) 23 wt. %	1	35	715	14.5	–	Bachman and Long, 2016
6FDA-durene	Ni_2_(dobdc) 21 wt. %	1	35	1,035	12.3	–	Bachman and Long, 2016
6FDA-durene	Mg2(dobdc) 50 wt. %	1	35	157	–	28	Smith et al., 2018
PDMS	Mg_2_(dobdc) 20 wt. %	2	25	2,100	–	12	Bae and Long, 2013
XLPEO	Mg_2_(dobdc) 10 wt. % wt. %	2	25	250	–	25	Bae and Long, 2013
PI	Mg_2_(dobdc) 10 wt. %	2	25	850	–	23	Bae and Long, 2013
Matrimid^@^	Ni(dobdc) 10 wt. % wt. %	1	25	2.58	1.9	–	Li W. et al., 2019
PIM-1	Mg(MOF-74) 10 wt. %	2	25	9,400	14.3	21.2	Tien-Binh et al., 2016
PIM-1	Mg(MOF-74) 15 wt. %	2	25	15,064	17.4	29.5	Tien-Binh et al., 2016
PIM-1	Mg(MOF-74) 20 wt. %	2	25	21,269	19.1	28.7	Tien-Binh et al., 2016

### MMMs Based on MOF Hybrids and Composites

It is worth to note that the controlled particle size of MOF crystallites can lead to better dispersion in the polymer matrix and result in defect-free membranes with high separation efficiency. However, the use of MOFs in MMMs still faces challenges such as the need for monodisperse particles and higher doping levels, and the risk of microvoid formation due to weak interfacial adhesion of the fillers with the polymer matrix (Lin et al., 2018). To mitigate these issues, researchers are focusing on the development of hybrid MOF fillers. Hybridizing MOFs with other materials including carbonaceous structures such as GO and CNTs results in new hybrid fillers that can exhibit strong dispersion within the polymer matrix, often increase the stability of the MOF counterparts, and create new porosity features and active sites, thus resulting in MMMs with a higher gas separation efficiency.

#### MOF/GO Hybrid Based MMMs

GO/MOF hybrids are often developed as fillers for fabricating defect-free MMMs with enhanced gas separation efficiency and multifunctionality. GO is known to have a high aspect ratio, which can play an essential role in increasing the selectivity of the resulting MMMs. The high aspect ratio of GO is responsible for increasing the path length, twists, and turns of the channels in MMMs, often allowing only small molecules to pass and restricting larger ones (Anastasiou et al., 2018). Of course, the absence of GO aggregates is a prerequisite for this behavior. Dong et al. reported the preparation of ZIF-8@GO hybrid via *in situ* growth of ZIF-8 on the surface of GO. This hybrid was incorporated into Pebax, and the effect of GO and ZIF-8 on the CO_2_ separation efficiency of the resultant MMM was studied. ZIF-8 filled PEBAX and GO incorporated PEBAX membranes were made as well, and their CO_2_ separation efficiencies as compared to the hybrid ZIF-8@GO based PEBAX membrane. It was observed that the CO_2_ permeability improved by 30% in ZIF-8/Pebax, mainly due to the presence of microporosity in the ZIF-8 itself. Moreover, ZIF-8@GO/Pebax showed a higher permeability (by 90%) and a selectivity of 1.7 relative to the pure Pebax due to the presence of GO along with ZIF-8 (Figures 4C,D) (Dong et al., 2016a).

Yang et al. (2019) incorporated ZIF-8@GO hybrid fillers into an ethylcellulose polymer matrix and studied its gas separation performance. The ZIF-8@GO formulation was prepared by the growth of ZIF-8 on the surface of GO through an ultrasonic synthesis method. Increasing the concentration of ZIF-8@GO increased the CO_2_ permeability of the MMM. The MMM with 15 wt. % of ZIF-8@GO hybrid filler showed the highest CO_2_ permeability of 203 Barrer and a CO_2_/N_2_ selectivity of 35 as compared to pure ethylcellulose membrane. Anastasiou et al. (2018) fabricated MMMs using PSF matrix and ZIF-8/GO hybrid filler through solution casting method and compared to pure PSF, and ZIF-8 filled MMM demonstrating that the ZIF-8/GO hybrid filler showed the highest performance in terms of both selectivity (CO_2_/N_2_ and CO_2_/CH_4_) and CO_2_ permeability. The intrinsic microporosity characteristics of the ZIF-8 component was responsible for the enhanced CO_2_ adsorption and increased CO_2_ permeability, while the GO component in the hybrid filler creates tortuous diffusion pathways in the MMM, allowing the small CO_2_ molecules to pass quickly and limiting the passage of larger gas molecules such as N_2_ and CH_4_, thus enhancing selectivity (Anastasiou et al., 2018).

Jia et al. synthesized hybrid UiO-66-NH_2_@GO nanoparticles *via* growth of UiO-66-NH_2_ on the surface of GO, and the hybrid nanoparticles were incorporated into a polyimide (PI) matrix to prepare MMMs. The MMM containing 5 wt. % of UiO-66-NH_2_@GO showed an enhanced CO_2_/N_2_ selectivity of 52 and CO_2_ permeability of 7 Barrer as compared to pure PI membrane (Jia et al., 2019). Feijani et al. prepared a new modified GO/MOF hybrid from CuBTC (copper (II) benzene-1,3,5-tricarboxylate) and GO nanosheets, and incorporated it into PVDF matrix, yielding hybrid MMMs with excellent mechanical and thermal properties, as well as enhanced performance in both selectivity and permeability (Feijani et al., 2018). Various filler concentrations were studied, and the optimum strength was found to be 9.5/0.5 wt. % of CuBTC/GO, resulting in the highest gas permeability and selectivity (Figure 4E). These positive effects are attributed to the filler morphology and uniform dispersion along with the excellent interaction with the polymer matrix (Feijani et al., 2018). Kang et al. prepared a set of thin and flexible MOF/GO composites with the support of Nylon matrix. HKUST-1 (copper-based MOF) was hybridized with GO through a layer-by-layer filtration method and was subsequently integrated into the polymeric membrane through *in situ* reaction with suitable cross-linkers. The as-fabricated membrane showed a H_2_/CO_2_ selectivity of 73 at 25°C and 1 bar (Kang et al., 2018). This approach can be extended to examine other MOFs combined with more flexible membranes. CO_2_ separation performance of the various MOF/GO based MMMs is shown in Table 5.

**Table 5:** CO_2_ separation performance of MMMs using MOF/GO hybrid fillers.

**Polymer/support**	**Filler (wt. %)**	**T (^**°**^C)**	**P (bar)**	**CO_**2**_ permeability (Barrer)**	**Selectivity**		**References**
					**Value**	**Relative to**	
Pebax	ZIF-8 @GO (6 wt. %)	25	1	249	47	CO_2_/N_2_	Dong et al., 2016a
Ethyl cellulose	ZIF-8 @GO (20 wt. %)	25	2	203	33	CO_2_/N_2_	Yang et al., 2019
PSF	ZIF-8/GO (5 wt. %)	25	2.5	1.7	5	CO_2_/N_2_	Anastasiou et al., 2018
PI	UiO-66-NH_2_@GO (5 wt. %)	25	3	7.28	52	CO_2_/N_2_	Jia et al., 2019
PVDF	CuBTC/GO (9.5/0.5 wt. %)	25	5	3.3	66.3	CO_2_/CH_4_	Feijani et al., 2018
Nylon (support)	HKUST-1@GO (N/A)	25	1	–	73	H_2_/CO_2_	Kang et al., 2018

#### MOF/CNT Hybrid Based MMMs

**Figure 5:**
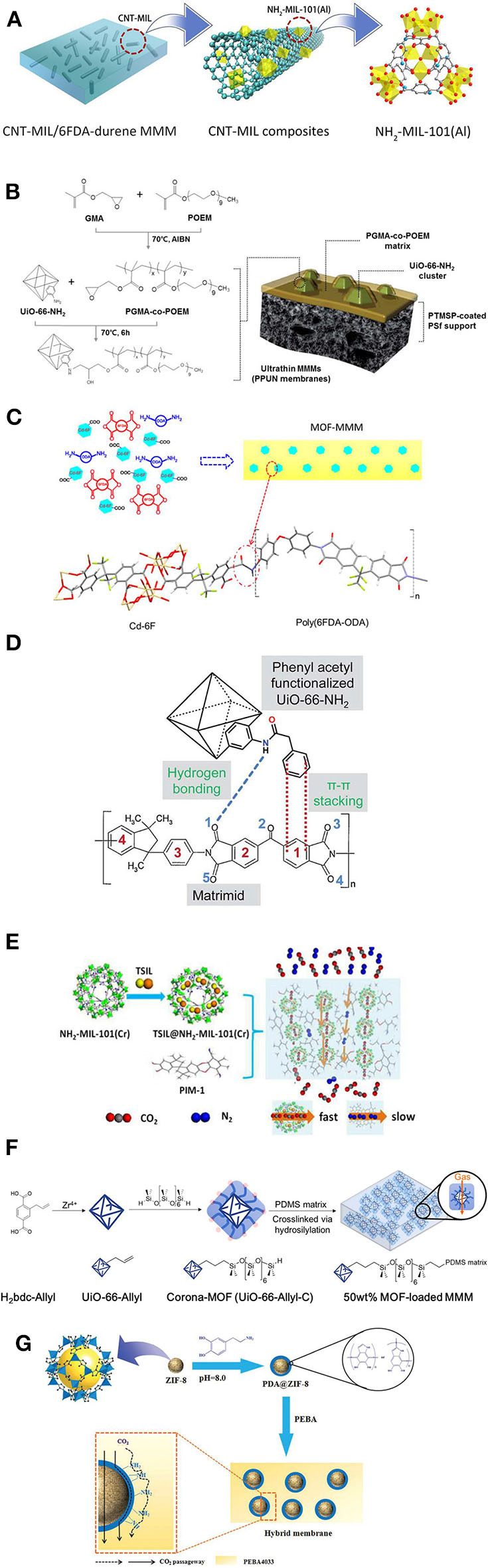
Schematic representations of characteristic examples of MOF MMM formation mechanisms and filler/matrix interactions: **(A)** NH_2_-MIL-101(Al)-decorated CNTs filled in 6FDA-durene membranes. Reproduced from Lin et al. (2015) with permission from ACS. **(B)** Synthesis of the PGMA-co-POEM copolymer and possible epoxide–amine reaction between UiO-66-NH_2_ and PGMA-co-POEM with illustration of ultrathin membranes. Reproduced from Kim et al. (2019) with permission from RSC. **(C)** Possible interaction between Cd-6F and 6FDA-ODA in MMMs upon *in situ* polymerization. Reproduced from Lin et al. (2014) with permission from ACS. **(D)** Possible interactions between the Matrimid® polymer and -PA modified MOFs. Reproduced from Venna et al. (2015) with permission from RSC. **(E)** Fabrication of TSIL@NH_2_-MIL-101 (Cr)/PIM-1 MMMs and the respective gas separation mechanism. Reproduced from Ma et al. (2016) with permission from RSC. **(F)** Formation of corona-MOFs and the respective corona-MOF loaded PDMS MMM. Reproduced from Katayama et al. (2019) with permission from ACS. **(G)** Formation of PDA@ZIF-8 and the possible CO_2_ transport mechanism in Pebax/PDA@ZIF-8 MMMs. Reproduced from Dong et al. (2016b) with permission from RSC.

To date, only a few articles report CNT-doped MOFs for MMMs preparation. Lin et al. enabled the effective separation of CO_2_/CH_4_ by CNT/MOF filled MMMs (Lin et al., 2015). The CNT/MOF composite was prepared by attaching NH_2_-MIL-101(Al) to the surface of CNTs and then incorporating it into the polyimide matrix to fabricate the MMMs (Figure 5A). A CNT/MOF hybrid containing CNTs modified with an additional amine group on the surface resulted in a strong adhesion with the polyimide matrix, even at the highest concentration of 15 wt. %. The CNT/MOF hybrid—filled MMMs exhibited both high CO_2_ permeability and high CO_2_/CH_4_ selectivity, while the optimum concentration of 5 wt. % of CNT/MOF hybrid led to CO_2_/CH_4_ selectivity of 29 and CO_2_ permeability of 780 Barrer. Sarfraz et al. developed MMMs by the simultaneous incorporation of ZIF crystals and CNTs into the polysulfone matrix and studied CO_2_ separation performance from humidified post-combustion gases. The hybrid fillers exhibited a synergetic effect in the MMM resulting in high CO_2_ permeability and CO_2_/N_2_ selectivity, while the MMM containing 18 wt. % ZIF-301 and 6 wt. % CNTs led to the most top separation performance with a CO_2_ permeability of 19 Barrer and CO_2_/N_2_ selectivity of 48 (Sarfraz and Ba-Shammakh, 2018).

### Functionalized MOFs in MMMs

Although various MOFs have been studied for the fabrication of defect-free MMMs, challenges remain such as control of interface voids and the aggregation of MOF crystals in the polymer matrix (Mahdi and Tan, 2016; Wang Z. et al., 2016). In this regard, functionalized MOFs or post surface-modified MOFs are employed to improve the interfacial interaction between MOFs and the polymer matrix, as well as to tune filler interaction with CO_2_ (Liu et al., 2018a,b,c). MOF-based MMMs have been prepared using functionalized MOFs and post-synthetic modified (PSM) MOFs through *in situ* and *ex-situ* methods. Different functional groups, including (-NH_2_) (Fu et al., 2017), sulfonic(-SO_3_H) (Rather and Siddiqui, 2019), fluorine(-F) (Fan W. et al., 2018), bromine (-Br) (Zhang et al., 2016), hydroxyl (-OH) (Dau et al., 2012), and vinyl (Satheeshkumar et al., 2018) have been employed. In general, the resulting MOFs show chemical and physical properties that are substantially distinct from pure MOFs. Most functionalized MOFs are developed using functional organic ligands through *in situ* approaches. It is worth noting that each functional group possesses different properties and reactivity. In particular, amine-functionalized MOFs have been extensively studied for acid gas separation due to their inherent basic properties (Pokhrel et al., 2018a) based on which they can easily interact with acidic gases and exhibit high adsorption capacity (Pokhrel et al., 2018b). For this reason, amine-functionalized MOFs have gained considerable attention for the fabrication of MMMs as well. Also, amine-functionalized MOFs possess a strong interaction with the polymer matrix, e.g., through hydrogen bonding, resulting in defect-free MMMs. Li et al. developed polyimide (PI) MMMs by incorporating different types of amine-functionalized MOFs (Jiang et al., 2019). Even a high loading of amine-functionalized MOFs (20 wt. %) showed a uniform dispersion and a strong bonding with the PI matrix, yielding defect-free MMMs. A 20 wt. % of NH_2_-MIL-53 doped PI membrane showed CO_2_ permeability of 2.8 Barrer and CO_2_/CH_4_ and CO_2_/N_2_ selectivity of 24 and 25, respectively, at 40°C and 3 bar (Jiang et al., 2019). Shen et al. fabricated MMMs using UiO-66-NH_2_ nanocrystals and incorporation of these in a polyether block amide (PEBA) matrix. The bonding between the MOF and the polymer matrix provided enhanced structural stability in addition to high CO_2_ permeability of 130 Barrer and CO_2_/N_2_ selectivity of 72 at for 10 wt. % of MOF loading at 20°C and 3 bar (Shen et al., 2016). Kim et al. fabricated MMMs using UiO-66-NH_2_ and complex poly-glycidyl methacrylate-co-poly (oxyethylene methacrylate) (PGMA-co-POEM) matrix by covalently binding the UiO-66-NH_2_ particles with the polymer matrix through a facile epoxide-amine reaction resulting in void-free MMMs exhibiting strong interfacial interaction (Kim et al., 2019). Moreover, increasing the UiO-66-NH_2_ content resulted in a dual pathway in the MMMs, which enhanced the gas permeability of the membrane (Figure 5B). The MMM containing 28.6 wt. % of UiO-66-NH_2_ nanoparticles displayed CO_2_ permeance of 488 GPU and a CO_2_/N_2_ selectivity of 32 at 25°C (Kim et al., 2019)

#### Incorporation of Functionalized MOFs Into MMMs via an “*in situ*” Process

*In situ*, polymerization is an effective process in which the MOFs are covalently cross-linked to the chains of the polymer matrix. The technique eliminates the formation of non-selective interfacial voids and the agglomeration of MOFs particulates in the MMMs. *In situ* polymerization is a convenient process for the introduction of MOFs into the polymer matrix because the polymer precursors easily self-distribute among the MOF crystallites resulting in controlled polymerization at the interface. Moreover, a strong and controlled interfacial interaction is achieved by this method. Lin et al. prepared MMMs by the incorporation of 4, 4′-(hexafluoroisopropylidene) diphthalic anhydride (6FDA) linked MOF crystals (Cd-6F) through *in situ* polymerization of the 6FDA-ODA polyimide. During this thermal polymerization, the presence of the uncoordinated-COO group on the Cd-6F surface interacts with the -NH_2_ groups of the ODA monomer at the terminal end of poly(6FDA- ODA) chains, yielding a defect-free MMM (Figure 5C). This material showed a CO_2_ permeability of 38 Barrer and CO_2_/CH_4_ selectivity of 45 in 2 bar pressure at 25°C (Lin et al., 2014).

Molavi et al. developed MMMs through *in situ* radical polymerization using methyl methacrylate with UiO-66, UiO-66-NH_2_ and vinyl group attached UiO-66. The *in-situ* polymerization provided substantial dispersion and interaction of UiO-66 nanoparticles within the PMMA matrix *via* covalent bonding. The polymer formation on UiO-66 nanoparticles reduced the agglomeration, yet resulted in non-selective interfacial voids in the MMMs. However, polymer grafting can partially block the porosity of the UiO-66, thus the gas separation often occurs only through a molecular sieving mechanism. Therefore, polymer grafted UiO-66 based MMMs showed a low CO_2_ permeability yet high CO_2_/CH_4_ (150) and CO_2_/N_2_ (110) selectivity due to the rigid interface in the MMMs (Molavi et al., 2018). Kumar et al. developed a MMM using vinyl-functionalized UiO-66, poly(ethylene glycol) divinyl ether (PEO-250), pentaerythritol tetra(3-mercaptopropionate) (PETM), 2,2′-(ethylenedioxy) diethanethiol (EDDT), and 2,2-dimethoxy-2-phenylacetophenone (DMPA) through an *in situ* thiol-ene photopolymerization. The vinyl-functionalized UiO-66 particles were covalently crosslinked with the polymer matrix through the C–S bond resulting in a strong adhesion of the MOF with the polymer matrix, yielding a void-free interface. Moreover, the photopolymerized MMM showed high flexibility and good mechanical strength. The 60 wt. % vinyl-functionalized UiO-66 MMM showed CO_2_ permeability of 467 Barrer and CO_2_/CH_4_ selectivity of 8 at 25°C and 5 bar (Satheeshkumar et al., 2018). Tien-Binh et al. fabricated MMM by *in situ* crosslinking of NH_2_-UiO-66 with a polymer possessing intrinsic microporosity (PIM-1). The *in-situ* polymerization yielded a high degree of interfacial coupling between the MOF and the polymer matrix. As a result, 20 wt. % of NH_2_-UiO-66 loaded MMM exhibited CO_2_ permeability of 12,498 Barrer with CO_2_/CH_4_ selectivity of 31.9 at 2 bar and 25°C (Tien-Binh et al., 2018).

#### Post-synthetically Modified MOFs in MMMs

Post-synthetic modification (PSM) is another strategy of modifying MOFs, in which organic functionalities and cross-linkers are grafted on the surface of the MOF particles to yield a functionalized material. PSM has certain advantages over the pre-functionalization method described in the previous section because a large number of functional groups can be incorporated into the MOFs without affecting the overall stability of the framework (Tanabe and Cohen, 2011). Amine-functionalized MOFs are frequently used for the PSM process because the amine groups, in addition to their affinity with CO_2_, they are highly reactive to other functional groups such as aldehydes, anhydrides, acyl halides, and epoxy groups thus enhancing the interaction of the funcltionalized MOFs with the polymer matrix. Selection of suitable functional groups depends on the chemistry of the polymer matrix. Post-modified MOFs are expected to possess a high dispersion and strong interfacial interactions with the polymer matrix, ensuing void-free MMMs.

Jiang et al. grafted UiO-66-NH_2_ with imidazole-2-carbaldehyde (ICA) through the PSM approach, and the modified MOF (UiO-66-NH2@ICA) was incorporated into a Matrimid® polymer (Jiang et al., 2019). The PSM increased the number of nitrogen atoms in the modified MOF *via* the imidazole group, which increased the dipole-quadrupole interactions between CO_2_ and nitrogen. The incorporation of 10 wt. % UiO-66-NH_2_@ICA into the Matrimid® polymer resulted in defect-free interfaces in the MMM, which exhibited a CO_2_ permeability of 40.1 Barrer and CO_2_/CH_4_ selectivity of 64.7 at 25°C and 3 bar (Jiang et al., 2019). Veena et al. modified the surface of UiO-66-NH_2_ using phenyl acetyl chloride (-PA) cross-linkers, decanoyl chloride, and succinic anhydride producing three different MOFs of aromatic-modified I (IPA), aliphatic C10-modified I (IC10), and acid-modified I (ISA) (Venna et al., 2015). The modified MOFs were incorporated into Matrimid^®^, and the surface-modified and unmodified MOFs showed nearly similar internal structure and functionality. However, the functional groups on the MOF surface influenced the interfacial adhesion between the polymer and the MOFs. Among the surface-modified MOFs, the -PA functionalized ones (IPA) showed a positive effect in the MMM performance, because of the enhanced π-π interactions between the aromatic groups in both the polymer and the phenyl acetyl (-PA) groups of the MOF structure. The bonding between the imide group of the polymer and the amine groups of IPA (Figure 5D) contributed positively as well. The MMM consisting of 23 wt. % of -PA surface-functionalized MOFs (IPA) exhibited CO_2_ permeability of 28 Barrer and CO_2_/N_2_ selectivity of 37 at 1 bar and room temperature (Venna et al., 2015). Zhu et al. post-modified the surface of MIL(53) using aminopropyltriethoxysilane (APTES), which was then incorporated into poly(ether imide) Ultem1000 to prepare high-quality asymmetric MMMs. The as-modified MIL-53 (S-MIL-53 (Al)) surface contained enriched amine groups, which enabled strong bonding with the amide groups of the polymer, thus enhancing the interfacial interaction with the matrix, favored dipole–quadrupole interactions with the CO_2_, and consequently increased the CO_2_ separation efficiency of the resulting MMMs. An asymmetric MMM consisting of 10 wt. % S-MIL-53 (Al) exhibited CO_2_ permeance of 24 GPU, which is 165% higher than that of the pure membrane (Zhu et al., 2016).

#### Ionic Liquid Modified MOFs in MMMs

In recent years, ionic liquids (ILs) were studied intensely for CO_2_ adsorption and separation processes due to their physicochemical properties such as high thermal and electrochemical stability, negligible volatility, and high CO_2_ solubility and selectivity (Perdikaki et al., 2012, 2016; Tzialla et al., 2013; Dai et al., 2016; Karousos et al., 2017, 2018). Based on these favorable properties, ILs have been applied as binder material for the fabrication of MMMs, which considerably improved the interfacial adhesion between filler/polymer and simultaneously enhanced the gas separation performance (Dai et al., 2016). Also, IL-based composite membranes have the potential of dual-action by combining capture and catalytic conversion performance (Perdikaki et al., 2012, 2016). In polymer-based MMMs however, the direct incorporation of ILs could reside in the free volume of the polymer matrix, thereby decreasing the gas diffusion properties of the resultant MMMs. Therefore, controlled inclusion of ILs is required during the fabrication of the MMMs. Grafting of ILs into the MOFs is one of the post-synthetic modification processes that could increase the interfacial affinity between the MOF and the polymer matrix. As a result, IL-modified MOF based MMMs can show high permeability and selectivity, mainly due to the high CO_2_ affinity of the ILs (Tzialla et al., 2013; Vicent-Luna et al., 2013; Ma et al., 2016). Lin et al. fabricated MMMs using the 6FDA-Durene polymer and an IL-coated HKUST. The layer of IL in MOF enhanced MOF-polymer interaction resulting in void-free MMMs with high CO_2_ permeability of 1101.6 Barrer and CO_2_/CH_4_ selectivity of 29.3 at 25°C and 2 bar (Lin et al., 2016). Li et al. studied CO_2_ selectivity and the mechanical properties of MMMs based on ZIF-8 modified by the ionic liquid of [bmim][Tf_2_N]. Incorporation of 15 wt. % of IL@ZIF-8 into Pebax matrix showed a significant increase in the tensile strength by ~20% and elongation at the break by 280%, which is higher than those of the non-modified membrane. Also, the MMM containing 15 wt. % of IL@ZIF-8 exhibited significant improvement in CO_2_ permeability to a value of 104.9 Barrer with CO_2_/CH_4_ selectivity of 34.8 at 25°C and 1 bar (Li et al., 2016). Ma et al. impregnated the task-specific ionic liquid (TSIL) of [C_3_NH_2_bim][Tf_2_N] on NH_2_-MIL-101(Cr) through a host-guest interaction approach. The MOF textural features enabled high dispersion of TSIL leading to the large active surface of the TSIL, which significantly enhanced the CO_2_ selectivity of a gas pair in the resulting MMMs. Furthermore, the NH_2_-MIL-101(Cr) contributed to the rapid CO_2_ transport and increased CO_2_ permeability (Figure 5E). The MMMs were prepared using different loadings of TSIL-modified NH_2_-MIL-101(Cr) in PIM-1 polymer, and 5 wt. % of TSIL-modified NH_2_-MIL-101(Cr) in the PIM-1 membrane resulted in CO_2_ permeability of 2,979 Barrer and CO_2_/N_2_ selectivity of 37 at 25°C and 3 bar (Ma et al., 2016). Interestingly, MOF-ionic liquid (IL) composite membranes have also been prepared with the MOF phase constituting most of the membrane volume. Specifically, ZIF-69 membranes were grown on porous α-alumina supports via seeded secondary growth and further functionalized by a CO_2_-selective tricyanomethanide anion/alkylmethylimidazolium cation-based ionic liquid (IL) to plug the gaps between the ZIF crystals yet leave the framework pores open for gas diffusion. The developed membranes at room temperature and under a 2 bar pressure exhibited CO_2_ permeance of 5.6 × 10^−11^ and 3.7 × 10^−11^ mol m^−2^ s^−1^ Pa^−1^ and CO_2_/N_2_ selectivity of 44 and 64 for CO_2_/N_2_ mixture, respectively (Tzialla et al., 2013).

#### Polymer-Coated MOFs in MMMs

A well-controlled interface between MOF/polymer phases could eliminate the non-selective defects in the MMMs and improve the gas separation performance. The interfacial affinity between the MOF/polymer phases depends to a large extent on the structure of the polymer matrix and the surface functionality of the MOFs (Guo et al., 2018; Jiang et al., 2018). Thus, carefully controlled modification of the polymeric phase on the MOF surface is needed for enhancing the permeability and selectivity of the resulting MMMs. Non-controlled polymer growth is unwanted as this may partially block the pores of the MOF, leading to permeability decrease (Li et al., 2006). Occasionally, weak interface adhesion is observed in few of the MOF-based MMMs, particularly involving micron-sized MOF fillers. Reducing the size of MOF particles can promote the interfacial surface area and increase the interfacial interactions (Li et al., 2005). Polymerization reactions such as radical, ionic, oxidative, and photopolymerizations have been employed to modify the surface of MOFs, often also resulting in the controlled growth of polymeric phase into the larger cavities of the MOFs (Zhang et al., 2015; Kitao et al., 2017). Wang et al. prepared covalently grafted polyimide brushes on the surface of various MOFs. These structures behaved as free-standing membranes at high loadings up to 88 wt. % of MOF even without incorporation of the additional polymeric matrix. The modified MMMs have shown high elasticity up to 472% due to a decrease in the matrix chain mobility and exhibited a significant plasticization resistance against CO_2_ as compared to the conventional MMMs. Also, the modified MMMs showed a simultaneous increase in both permeability and selectivity for CO_2_/N_2_ and CO_2_/CH_4_ separation (Wang H. et al., 2018). Katayama et al. modified allyl terminated UiO-66 with hydride-terminated poly (dimethylsiloxane), yielding a PDMS-coated MOF (“corona-MOF”). 50 wt. % of corona-MOF particles were subsequently incorporated to the PDMS matrix to fabricate a defect-free MMM (Katayama et al., 2019). Corona-MOF based MMMs possess a robust covalent interaction between both MOF and the polymer phases leading to negligible particle aggregation during the film curing and resulting in free-standing, flexible MMMs of <1 μm thickness (Figure 5F). Moreover, corona-MOF based MMMs did not exhibit any undesired formations such as plugged sieves and sieve-in-a-cage ones, and showed high CO_2_ permeability (4,859 ± 727 Barrer) without loss in selectivity (CO_2_/N_2_ of 10) at 1 bar (Katayama et al., 2019).

Dong et al. developed MMMs using a Pebax matrix with different loadings of polydopamine (PDA)—coated ZIF-8 (Dong et al., 2016b). The PDA coating could eliminate the interfacial defects in the MMM and enhance the interfacial adhesion between ZIF-8 and the polymer matrix. The permeability and selectivity of MMMs often vary in humid or dry conditions, a fact that should be taken into consideration when it comes to practical applications. In humid conditions, the amine groups in PDA@ZIF-8 acted as CO_2_ adsorption sites at the polymer/MOF interface. The CO_2_ molecules pass through the membrane *via* both a transport mechanism and a solution-diffusion mechanism (Figure 5G). A 15 wt. % of PDA@ZIF-8 Pebax membrane showed a CO_2_ permeation of 267 Barrer and a CO_2_/N_2_ selectivity of 62 at 1 bar and 25°C in humid conditions. At dry conditions though, the CO_2_ permeation follows only a solution-diffusion mechanism resulting in a decrease in CO_2_ permeability (220 Barrer) and CO_2_/N_2_ selectivity (56) at the same pressure and temperature (Dong et al., 2016b). Guo et al. modified the surface of CAU-1 nanoparticles using polyethyleneimine (PEI) with glutaraldehyde (GA) as an intermediate covalent cross-linker. As-modified CAU-1 (PEI-CAU-1) nanoparticles were incorporated into poly (ethylene glycol) diacrylate to fabricate MMM through a photopolymerization reaction. The enriched amine groups in the PEI-CAU-1 acted as CO_2_ carriers favoring the CO_2_ transport through the MMMs. The PEI functionalization also reduced the MOF aggregation in the MMM. An MMM consisting of 30 wt. % of PEI-CAU exhibited CO_2_ permeability of 546 Barrer and CO_2_/CH_4_ selectivity 27.8 at 35°C and 3 bar (Guo et al., 2018).

## The Trade-Off Curves of MOF-Based MMMs for CO_2_/CH_4_ and CO_2_/N_2_ Separation

**Figure 6:**
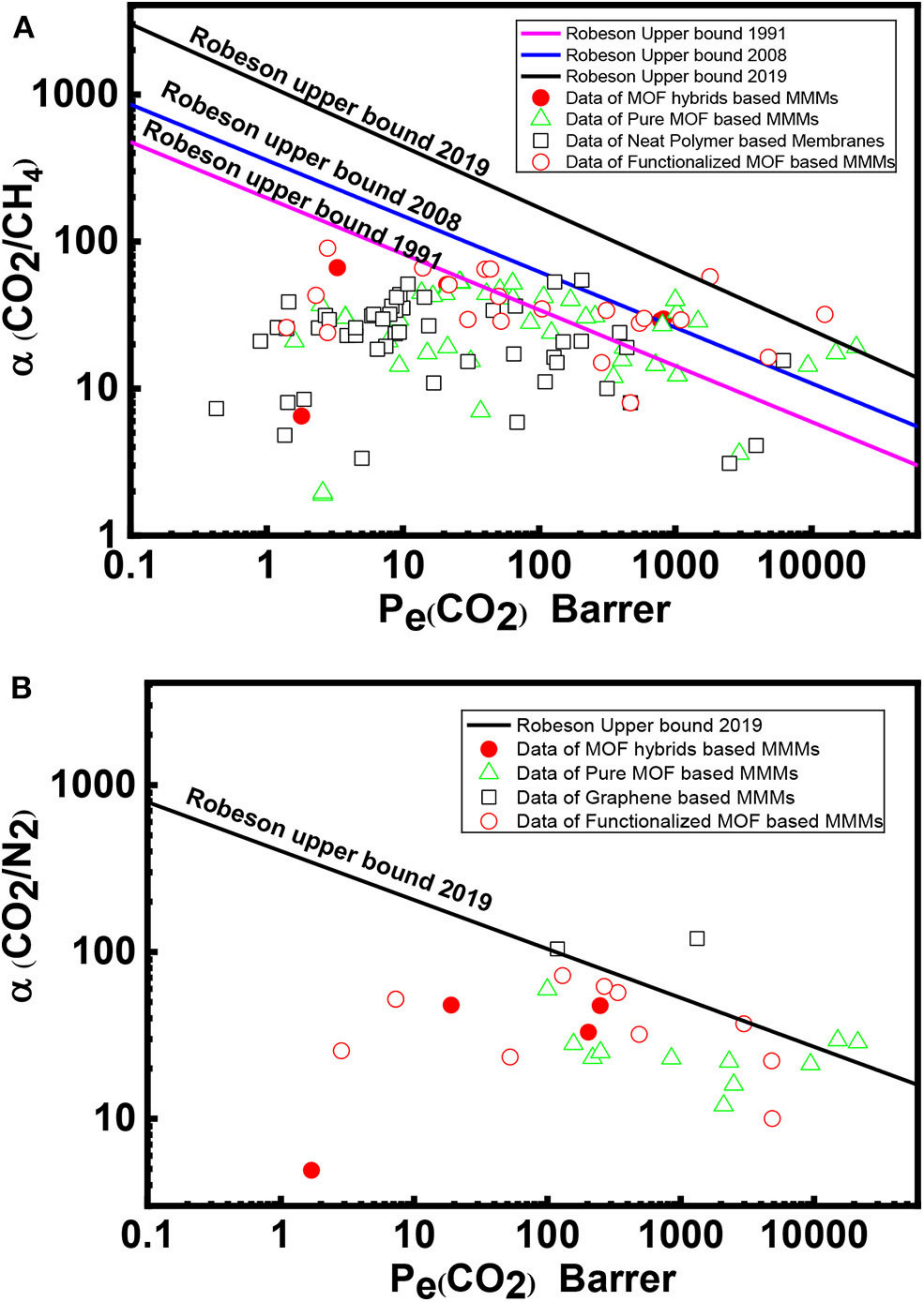
The performance of MOF-based MMMs [HKUST-1 (Car et al., 2006; Hu et al., 2010; Ge et al., 2013; Lin et al., 2016; Feijani et al., 2018; Chi et al., 2019); ZIF-7 (Li et al., 2013; Azizi and Hojjati, 2018); ZIF-8 (Ordoñez et al., 2010; Ban et al., 2015; Shahid et al., 2015; Dong et al., 2016a,b; Jusoh et al., 2016; Lin et al., 2016; Anastasiou et al., 2018; Castro-Muñoz and Fíla, 2019; Yang et al., 2019; Zheng et al., 2019); UiO-66 (Nik et al., 2012; Dong et al., 2016b; Shen et al., 2016; Satheeshkumar et al., 2018; Sutrisna et al., 2018; Tien-Binh et al., 2018; Zamidi Ahmad et al., 2018; Jia et al., 2019; Jiang et al., 2019; Katayama et al., 2019; Kim et al., 2019); MOF-5 (Perez et al., 2009); MIL-53 (Zornoza et al., 2011a; Chen et al., 2013; Hsieh et al., 2014; Ahmadi Feijani et al., 2015; Feijani et al., 2015; Tien-Binh et al., 2015; Mubashir et al., 2018b; Jiang et al., 2019); ZIF-90 (Bae et al., 2010); ZIF-11 (Safak Boroglu and Yumru, 2017); KAUST-7 (Chen K. et al., 2018); CuBDC (Cheng et al., 2017); Mg-MOF-74 (Bae and Long, 2013; Tien-Binh et al., 2016; Smith et al., 2018); Ni-MOF-74 (Bachman and Long, 2016; Yang et al., 2019); ZIF-94 (Etxeberria-Benavides et al., 2018); MIL-101 (Xin et al., 2015; Ma et al., 2016); Bio-MOF-1 (Ishaq et al., 2019); Fe(BTC) (Nabais et al., 2018)] included in this review work for **(A)** CO_2_/CH_4_, and **(B)** CO_2_/N_2_ separation data in conjunction to redefined Robeson upper bounds (2019).

Robeson first reported the general trade-off between permeability and selectivity for polymeric membranes in 1991 (Robeson, 1991), which was further updated with new data in 2008 (Robeson, 2008). The Robeson trade-off curves provide a direction to the researchers toward the development of new efficient membranes to surpass the existing permeability and selectivity performances for common gas pairs. A primary goal of MMMs prepared in a combination of a polymer matrix with various fillers is to achieve better perm-selectivity and surpass the current Robeson trade-off curve. Redefining the Roberson upper bound in 2019 (Comesaña-Gándara et al., 2019) set new aspirational targets for the membrane community to aim more attractive parametric optimization of performance, energy use, and cost-efficiency of the CO_2_ separation process. The Robeson trade-off line is represented by $P_{i}=k\alpha_{ij}^{n}$, where *P*_*i*_ is the permeability of the more mobile gas, α_*ij*_ is the selectivity of component *i* over component *j, k* is the constant factor, and *n* is the slope of the log-log graph of the above equation. The Robeson trade-off line parameters for the CO_2_/CH_4_ gas pair is shown in Table 6. The permeability and selectivity values for CO_2_/CH_4_ and CO_2_/N_2_ separation using MMMs based on this review along with the redefined Robeson upper bounds of 2019 are shown in Figures 6A,B. The data provide an insight into the MMMs development from 1998 to the present. It can be noticed that MMMs based on MOF composite/hybrid fillers as well as on functionalized MOFs show better performance and some of these cross the 2019 redefined Roberson upper bound, as presented in Table 7. As a characteristic example, CNT/MOF composite fillers prepared by attaching NH_2_-MIL-101(Al) to the surface of CNTs and then incorporated in polyimide showed CO_2_ permeability of 818 Barrer with CO_2_/CH_4_ selectivity of 29.7 (Lin et al., 2015). In addition, MMMs fabricated by *in situ* crosslinking of NH_2_-UiO-66 with a polymer of intrinsic microporosity (PIM-1) exhibited a high degree of the interfacial binding between the MOF and the polymer matrix. As a result, 20 wt. % of UiO-66-NH_2_ MMM showed CO_2_ permeability of 12,498 Barrer with CO_2_/CH_4_ selectivity of 31.9 at 2 bar and 25°C (Tien-Binh et al., 2018). Hence, MMMs comprised of MOF hybrid and functionalized fillers in a suitable polymer matrix constitute promising perm-selectivity membranes for CO_2_ abundant streams.

**Table 6:** Robeson trade-off line parameters for CO_2_/CH_4_.

	***k* (Barrer)**	***N***	**References**
Robeson Upper bound 1991	1,073,700	−2.626	Robeson, 1991
Robeson Upper bound 2008	5,369,140	−2.636	Robeson, 2008
Redefined Robeson Upper bound 2019	22,584,000	−2.401	Comesaña-Gándara et al., 2019

**Table 7:** MOF—based MMMs with superior performance.

**Polymer**	**Filler**	**P (bar)**	**T (^**°**^C)**	**Gas pair**	**CO_**2**_ permeability (Barrer)**	**Selectivity CO_**2**_/CH_**4**_**	**Selectivity CO_**2**_/N_**2**_**	**References**
Polymer of intrinsic microporosity (PIM-1)	20 wt. % NH_2_-UiO-66	2	25	CO_2_, CH_4_, N_2_ pure gases	12,498	31.9	54.2	Tien-Binh et al., 2018
6FDA-Durene Polymide	5 wt. %. CNT-MIL	2	25	CO_2_/CH_4_ (50/50%)	818	29.7	–	Lin et al., 2015
PIM-1	TSIL-modified NH_2_-MIL-101(Cr) (5 wt. %)	3	25	CO_2_, N_2_ pure gases	2,979	–	37	Ma et al., 2016
Pebax MH 1657	10 wt. % PEG–PEI–GO	2	25	CO_2_/N_2_ (10/90%) CO_2_/CH_4_ (30:70%)	1330	45	120	Li et al., 2015
Pebax	6 wt. % ZIF-8@GO	1	25	CO_2_, N_2_ pure gases	249	–	47.6	Dong et al., 2016a
PIMs	MOF-74 (20 wt. %)	2	25	CO_2_, N_2_, CH_4_ pure gases, and CO_2_/CH_4_ mixture (mole ratio of 1:1)	21,269	19.1	28.7	Tien-Binh et al., 2016

Comesaña-Gándara et al. redefined the Robeson upper bound in 2019 for the CO_2_/N_2_ separation with k and n values of 755,580,000 and −3.409, respectively (Comesaña-Gándara et al., 2019). The permeability and selectivity for the CO_2_/N_2_ gas pair based on this review along with 2019 redefined Robeson upper bound are shown in Figure 6B. Notably, TSIL-modified NH_2_-MIL-101(Cr) loaded PIM-1 MMM showed a CO_2_ permeation of 2,979 Barrer and a CO_2_/N_2_ selectivity of 37 (Ma et al., 2016). As a comparison, functionalized graphene-based MMMs can cross or exceed the upper bound. For instance, PEG–PEI–GO (10 wt. %) loaded in Pebax MH 1657 membrane exhibits CO_2_ permeability of 1,330 Barrer and CO_2_/N_2_ selectivity of 120 (Li et al., 2015). Efforts need to continue to further optimize MOF-based MMMs according to the nature of the polymer matrix and the structure of the MOFs paying attention to filler-matrix interaction, processing methods, membrane thermal and mechanical stability and including innovative fabrication of functionalized MOFs and hybrid MOF systems. Designing new MOF-based nanoparticle combinations as fillers could be a promising approach for the development of MMMs to surpass the Robeson upper bound limits.

## Perspectives on MMMs and MOF-Based MMMs for Large-Scale Integration

The integration of membranes in commercial applications has taken off in the last decade. For instance, cellulose acetate membranes tend to become the standard practice in the Far East for the removal of CO_2_ from natural gas. These applications, mostly off-shore, aim to remove CO_2_ from high-pressure streams (40–70 bar) from typical 10–30% CO_2_ to pipeline specification (~2–5% CO_2_), which is regarded as bulk separation. The main advantage of membrane separation over conventional processes is that the membranes do not require bulky columns and heat for regeneration in contrast to the traditional amine absorption processes. Overall, this provides an economic incentive to utilize membranes. Two leading membrane suppliers are UOP and Schlumberger (formerly NATCO) who employ improved versions of celluloses acetate membranes. Another high-pressure application under consideration is the removal of CO_2_ from synthesis gas (H_2_ and CO at 15–30 bar), but no related commercial applications are known to the authors. Recently, there has been a considerable interest to apply membranes also for low-pressure flue gas streams. This means that the separation is from nitrogen-rich streams at very high volumetric flowrates and likewise, that the demand on the membrane performance becomes even higher to make such an application economically feasible.

In this paper, developments of a specific type of membranes (MOF-based MMMs) are reviewed. Membrane performance is generally expressed to permeability and selectivity, yet it should be realized more factors are dictating the overall economics of a membrane process. Most importantly, it should be appreciated that the separation is based on the difference in partial pressure of the component over the membrane and this driving force is a limiting factor, either because the partial pressure in the inlet gas is low, or a very tight specification (<1 bar partial pressure) should be met. For high-pressure streams, it means that the application is restricted to bulk separation. For low-pressure streams, it means that compression must be applied at the retentate side or vacuum at the permeate side and this is often an inhibiting factor for the economics of the process.

In general, the permeance will determine the required membrane surface area. Higher permeance leads to lower membrane areas and thus more economical cost. The selectivity will evaluate the losses of the component that should not be permeating. In the case of natural gas treatment, lower selectivity leads to higher loss of methane to the CO_2_ stream, which is an economic loss. This can be unusually large at high partial pressures of CO_2_. It should be realized that polymeric membranes tend to lose a significant fraction of their selectivity at high partial pressures of components like CO_2_ (also H_2_S) due to a plasticization. So, the performance should be evaluated at the appropriate partial pressure. The selectivity with respect to N_2_ is less of an issue as loss of N_2_ has no value consequence, while the permeating CO_2_ does not have to be ultra-pure. Indicatively, it can contain up to 2% of inerts for enhanced oil recovery (EOR) purposes.

Another limiting factor to selectivity is the pressure ratio between inlet P_0_ and permeating stream P-_L_. For instance, the relation trend between permeate vapor concentration and the pressure ratio (P_o_/P_L_) indicates that increasing the selectivity above the pressure ratio is not useful. For high-pressure systems, this means that a selectivity above 50 is not entirely useful (inlet pressure 50–60 bar, outlet 1 bar, pressure ratio 50). When CO_2_ must be injected, it is beneficial to have the CO_2_ permeating at elevated pressure. This will limit the flux to some extend because it depends on the absolute pressure difference (P_0, CO2_-P_L, CO2_) only. The pressure ratio will be strongly affected though. When P_L_ is increased to 4 bar the pressure ratio may go down to around 10, and it is not useful to have a selectivity above this value. For flue gas treatment, the pressure ratio is the most important factor determining the process because both compression and vacuum are needed. As an example, with compression up to 3 bar and vacuum of 0.1 bar at the permeate side the pressure ratio is 30. These are the values proposed by the leading party in this field, MTR. MTR aims a permeance of 1,500 GPU and a selectivity of 25–30 (Baker et al., 2018). This means optimizing flux, and thus permeance is more important than improving selectivity.

The membrane process providers have developed process flow schemes that have improved characteristics of the overall process, such as multi-stage membrane systems, recycle options, and sweep gas. This may influence the optimal parameters of the membrane. To establish that, detailed process design and sensitivity studies need to be executed. However, this will not influence the major conclusion, i.e., that permeance is the most critical factor. Based on this review and the Robeson plots of Figure 6, the most promising systems are the MOF hybrids, functionalized MOFs, and graphene-based MMM systems. Currently, the selectivity is considered well within what is required, so it is advised to look at systems with higher flux, even at a lower selectivity (25–50). It should be noted that polymeric membrane systems are also improving, which means that only time will tell which system will be the winner.

Finally, it should be realized that other factors determine the success of a membrane system as well such as the ease of processing, potting, the lifetime, and resistance to contaminants. Especially the latter requires particular attention and determines the need for pretreatment or heating of the gas flow. That is beyond the purpose of this review, but generally, it requires extensive development. Sometimes these issues only emerge in field trials.

## Summary and Outlook

Applications of MMMs in gas separation have attracted great attention in recent years. In this review, through selected examples we analyze the advances made on MOF-based MMMs for CO_2_ separation. The review provides an overview of the merits of MOF MMMs compared to the polymeric and inorganic membranes, and it documents that MOF-based MMMs show a promising performance and have the potential to overcome the traditional permeability and/or selectivity trade-off barriers. The significance of MOFs on the development of MMMs is highlighted in conjunction with the redefined Robeson upper bounds (2019). The current reports reveal that MOFs are attractive fillers offering unique performance features to the resulting MMMs in terms of high permeability combined with enhanced selectivity as long as the fillers, matrix, and the interaction between these two phases are properly configured. The size, structure, porosity, and nature of organic ligands in the MOFs also play a vital role in the effective dispersion within the polymer matrix, in the separation behavior, as well as in enhancing the membrane robustness.

Interface engineering and surface functionalization can improve adhesion and yield defect-free formulations. Indeed, at high filler loadings in particular, voids may develop resulting in reduction in separation performance. This issue can be addressed by functionalization of the MOF surfaces thus offering a multiple benefit of strong interfacial adhesion between MOFs and the polymer, better dispersion and prevention of agglomeration of the MOF crystallites in the MMM, and tuning of the transport behavior of the permeating gas molecules. In addition, modification of the internal pore surface of the MOF fillers needs to be further investigated. The confinement of organic species into the nanocages of MOFs, e.g., via *in-situ* or wet post-growth process should be carried out systematically. Such structure can provide advanced porous MOF characteristics and tunable filler performance within the membrane matrix for targeted separations, which combined to the intrinsic pore merits can manipulate diffusion behavior and enhance molecular retention, diffusion, and separation (Smaldone et al., 2010; Hartlieb et al., 2016). Significant attention and promising performance are also associated with hybrid fillers that offer multifunctionality thus serving more targets and specifications compared to mono-fillers. For example, graphene oxide, CNTs, zeolite and silica nanoparticles are often combined with MOFs to tune the permeability, selectivity, filler dispersion, porosity, and the interfacial properties in the resulting MMMs. For instance, in MOF/GO hybrid—based MMMs, MOFs can enhance permeability and GO can enhance selectivity thus both targets are being met.

Overall, MOFs are suitable fillers in MMMs, yet there is still ample room for continued research particularly on functionalization and hybridization of these structures to meet the targets of high permeability and selectivity, good mechanical, chemical, and thermal stability, scalability, and low cost, thus paving the route for the MOF-based MMMs to deliver on their promises in today's demanding CO_2_ separation needs.

## Author Contributions

All authors contributed in the collection of data, critical evaluation, and writing of this review.

## Conflict of Interest

FG was employed by the company ADNOC Gas Processing. The remaining authors declare that the research was conducted in the absence of any commercial or financial relationships that could be construed as a potential conflict of interest.
